# An interpretable alphabet for local protein structure search based on amino acid neighborhoods

**DOI:** 10.1093/bioinformatics/btaf458

**Published:** 2025-08-23

**Authors:** Saba Zerefa, Jesse Cool, Pramesh Singh, Samantha Petti

**Affiliations:** School of Engineering and Applied Sciences, Harvard University, Cambridge, MA 02138, United States; Department of Mathematics, Tufts University, Medford, MA 02155, United States; Tufts Institute for Artificial Intelligence, Tufts University, Medford, MA 02155, United States; Department of Mathematics, Tufts University, Medford, MA 02155, United States

## Abstract

**Motivation:**

Recent advancements in protein structure prediction methods have vastly increased the size of databases of protein structures, necessitating fast methods for protein structure comparison. Search methods that find structurally similar proteins can be applied to find remote homologs, study the functional relationships among proteins, and aid in protein engineering tasks.

**Results:**

We design a “3Dn” structural alphabet that encodes the local neighborhoods around each amino acid in an interpretable way. In a search benchmark task, a combination of our alphabet and Foldseek’s 3Di alphabet, outperforms each alphabet individually and ranks best among local search methods that do not require amino acid identity information. We provide software tools that enable the exploration of novel alphabets and combinations of alphabets for protein structure search.

**Availability and implementation:**

The code is freely available at https://github.com/spetti/structure_comparison and at Zenodo https://doi.org/10.5281/zenodo.15734371.

## 1 Introduction

Recent developments in protein structure prediction methods have resulted in dramatic increases in the size of protein databases, with AlphaFold alone providing predicted structures for each of the over 250 million proteins in UniProt as of 2025 (UniProt Consortium 2015, Jumper *et al.* 2021). The newly expansive size of protein databases demands fast and computationally efficient algorithms for high quality protein structure analysis. Search methods that find structurally similar proteins can be applied to find remote homologs, study the functional relationships among proteins, and aid in protein engineering tasks. Structural alignment algorithms like TM-align and Dali effectively compare protein structures based on their 3D configurations; however, these algorithms are too slow and computationally expensive for searching large databases (Zhang and Skolnick 2005, Holm 2020).

In contrast, classic sequence comparison methods utilize dynamic programming (Altschul *et al.* 1990, Finn *et al.* 2011, Hauser *et al.* 2016) to efficiently search databases with hundreds of millions of proteins. Traditionally, these methods only compare primary sequences. The authors of Foldseek (Van Kempen *et al.* 2024) designed an alternate alphabet that encodes information about the 3D structure of the protein. By using this alphabet within established highly optimized search software designed for amino acid sequences, they created a tool for structure comparison search that is tractable for large databases without a large drop in accuracy as compared to Dali and TM-Align (Zhang and Skolnick 2005, Holm 2020).

Here, we focus on designing an alternate structural alphabet for structure comparison search. Foldseek uses a neural network with a VQ-VAE (Vector Quantized Variational Autoencoder) architecture to learn an alphabet of twenty 3Di characters. The 3Di characters encode the relationship between an amino acid and its nearest amino acid neighbor nonadjacent in sequence. Due to the black-box nature of the machine learning components involved in training, the 3Di characters are difficult to interpret, making it challenging to extract biochemical meaning of any given 3Di character. Additionally, only using the single nearest neighbor to an amino acid when constructing 3Di characters limits the amount of structural information captured by the 3Di characters. The nearest neighbor method may be sensitive when comparing two closely related proteins if there are multiple amino acids at a similar distance. An alphabet encoding richer features has the potential to improve the accuracy of the search, while leaving the remainder of the search algorithm unchanged. Thus, the construction of alternate alphabets merits further investigation.

We develop interpretable methods that encode the local structure around an amino acid by taking into account the locations and secondary structure of nonadjacent neighbors within a fixed radius around the amino acid. By considering all neighbors within a fixed radius, we are able to capture local structure information beyond just a single neighbor, and by focusing on interpretable methods, we are able to extract biochemical meaning from our alphabet characters. We compare our alphabet, which we call 3Dn (*n* for “neighborhood”) to Foldseek’s 3Di alphabet on a protein search benchmarking task. We also compare combinations of these alphabets with the amino acid alphabet with BLOSUM62 scoring and an alphabet derived from backbone dihedral angles. Alphabet combinations that do not require amino acid identities may be of particular interest for searching when homology is not expected, e.g. for protein engineering applications or in cases of convergent evolution.

### 1.1 Summary of contributions

Our results can be summarized as follows:

- As a precursor to our “3Dn” alphabet, we first introduce *blurry neighborhoods* to describe the locations of nonadjacent neighbors of an amino acid within 15Å. We devise an accompanying Jaccard metric to score the similarity of two blurry neighborhoods. Using this scoring scheme, we achieve significantly improved performance on a structure search task as compared to search with Foldseek’s 3Di alphabet, and comparable results with the combination 3Di and amino acid alphabet.
- We introduce the 3Dn alphabet, built by clustering the blurry neighborhoods. The 3Dn alphabet performs marginally worse than the 3Di alphabet on a protein database search task, but combining the 3Dn and 3Di alphabets improves on the performance of each individual alphabet. The combined 3Di-3Dn alphabet has comparable performance to the slower blurry neighborhood approach and is the current state of the art alphabet combination for local search that does not require amino acid identity information.
- We visualize the neighborhoods encoded by each 3Dn character. Then, we analyze the co-occurrence of 3Di and 3Dn characters with each other, amino acid identity, and an alphabet of backbone dihedral angles built by a mutual information clustering algorithm. We conclude that the 3Dn and 3Di alphabets are capturing different aspects of the conserved structure, thus explaining the improved performance of the combined alphabet.
- We establish a framework for exploring new structural alphabets. In addition to an efficient implementation of the search benchmarking task, we provide code to compute BLOSUM-like substitution matrices, combine multiple alphabets, and reduce alphabet size via clustering based on mutual information. The software is independent of the type of structure information used, and thus can be used with other structural alphabets.

### 1.2 Related work

Protein structure comparison methods experience tradeoffs with respect to algorithm efficacy, computational efficiency, and interpretability. Algorithms like TM-align and Dali are accurate (Zhang and Skolnick 2005, Holm 2020), yet the computational expense of such techniques makes them infeasible for large protein database searching tasks. Works such as AlphaFind (Procházka *et al.* 2024), PLMSearch (protein language model) (Liu *et al.* 2024), and DHR (dense homolog retriever) (Hong *et al.* 2025) utilize deep learning techniques to construct an embedding for the whole protein and infer similarity by comparing embeddings. While these methods allow for efficient global search, they are not effective for local search in which only parts of the protein are conserved.

Here, we focus on developing an alphabet that can be used with existing local search tools. We compare against the aforementioned Foldseek 3Di alphabet, which was trained with a VQ-VAE architecture. Recent work trained an alternate alphabet and accompanying scoring scheme in an end-to-end manner using message-passing architecture of ProtMPNN (Dauparas *et al.* 2022, Trinquier *et al.* 2025). In contrast, we specifically focus on deriving an alphabet from explicitly defined features to arrive at an interpretable, black-box free alphabet.

Early examples of protein structure alphabets encode *sequentially* local structure; the alphabet character assigned to a particular residue is determined by the substructure of the protein containing the residues within a few positions in the sequence (Ma and Wang 2014). The kappa-alpha alphabet of Tung *et al.* (2007) is derived from angles between sequentially nearby $C_{\alpha }$ atoms. Other approaches identify structural motifs formed by groups of consecutive residues (Rooman *et al.* 1990, Bystroff and Baker 1998, de Brevern *et al.* 2000, Camproux *et al.* 2004, Le *et al.* 2009). Our alphabet based on backbone dihedral angles also models sequentially local structure and is similar to the kappa-alpha alphabet (Tung *et al.* 2007). However, our dihedral alphabet is derived from different angle measurements, and we use a mutual information based clustering algorithm to discretize the space of angle pairs, which in our context is the Ramachandran plot. An alternate approach for local alignment based on pairs of backbone dihedral angles does not discretize Ramachandran plot and instead defines a score between pairs of backbone angles based on a geometric notion of distance (Miao *et al.* 2008) that does not account for observed patterns of conservation in evolutionarily related proteins. In contrast the aforementioned methods, our 3Dn alphabet captures *spatially* local structure; the structure character assigned to a residue is influenced by spatially nearby residues that may be distant in the sequence.

Works such as Heinzinger *et al.* (2024) and Johnson *et al.* (2024) train neural networks to predict the Foldseek 3Di characters from the protein primary sequence, thus enabling structural search without access to a predicted structure. The same methods could be applied to predict any alphabet or combination of alphabets from the primary sequence.

## 2 Materials and methods

### 2.1 Representing local amino acid neighborhoods

We quantify similarity between a pair of amino acids in different structures by comparing the locations and secondary structures of the amino acids in close proximity to them. We consider two amino acids of the same sequence to be *neighbors* if their alpha carbons are within 15Å of each other, and they are >5 positions away in the sequence.

**Figure 1.**
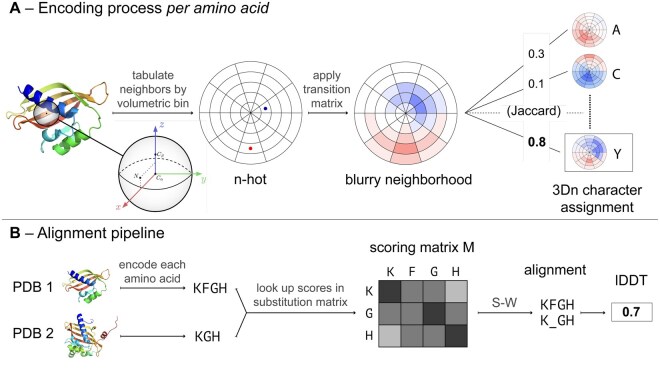
Overview of the process for encoding a 3Dn sequence and generating an alignment. (A) Encoding process for a single amino acid. We define a reference frame centered at the $C_{\alpha }$ atom and divide the surrounding 15Å sphere into bins. Then we compute an *n*-hot vector that tabulates the positions and secondary structures of neighboring amino acids in the sphere. The *n*-hot vector shown is a simplified 2D example. A transition matrix is applied to the *n*-hot vector, resulting in a blurry neighborhood. The blurry neighborhood is compared via weighted Jaccard similarity to twenty landmark blurry neighborhoods, each of which are affiliated with a 3Dn character. The amino acid is assigned to the 3Dn character with the largest weighted Jaccard similarity value. (B) Pipeline demonstrating how to obtain an alignment from two PDB files. Each amino acid is encoded into a 3Dn character according to the process outlined in A. For simplicity, we only show a short (three or four character) subsequence of the encoded sequence. A scoring matrix for the pair of encoded sequences is generated by looking up a substitution score for each pair. With this scoring matrix, the Smith-Waterman (S-W) algorithm is used to generate an alignment, the quality of which is computed using lDDT. Protein depictions are courtesy of SCOPe (Chandonia *et al.* 2019).

First, we designed a common reference frame to compare the relative positions of the neighbors between two proteins. The reference frame is defined with respect to an individual amino acid and is independent of rotation or translation of the greater protein. The reference frame of an amino acid satisfies three rules: the alpha carbon lies at the origin, the vector between the alpha carbon and beta carbon lies on the positive *z* axis ^1^, and the nitrogen lies on the *XZ* plane, with positive *X* coordinate. A visualization of the reference frame is provided in Fig. 1A. We discretize the sphere with radius 15Å into 250 sections of equal volume using spherical coordinates. Note that the 15Å sphere consists of all points (R,θ,φ) such that R∈[0,15Å],  θ∈[0,π], and φ∈[0,2π]. We partition the interval [0,15Å] into 5 subintervals, the interval [0,π] into 5 subintervals, and the interval [0,2π] into 10 subintervals, to acquire the 250 bins of equal volume. For an explicit formulation of the subintervals, see Appendix A.1, available as supplementary data at *Bioinformatics* online. We assign each neighbor to one of 1000 “bins” determined by which of the 250 sections of the sphere the neighbor occupies and the secondary structure of the neighbor: beta sheet, left helix, right helix, or other, as determined by the backbone dihedral angles (see Appendix A.1, available as supplementary data at *Bioinformatics* online).

For each amino acid, we define an *n -hot vector* $\vec{v}$ encoding the locations and secondary structure of neighbors: $\vec{v}\in {\left(Z_{\geq 0}\right)}^{B},$ where *B* is the number of bins. The value $v_{i}$ represents the number of neighbors of amino acid *x* that are assigned bin *i*.

#### 2.1.1 Blurry neighborhoods

Our goal is for amino acids with similar *n*-hot vectors to be assigned the same structural character. Although *n*-hot vectors capture the local structure around an amino acid, their discrete nature poses challenges in comparing two different *n*-hot vectors. For instance, between two structurally similar proteins, the bin of an analogous neighbor may not be completely conserved but rather moved to an adjacent bin.

We address these limitations by introducing the blurry neighborhood, which is a continuous vector that builds upon the notion of the *n*-hot vector by capturing empirical patterns of how neighborhoods differ between structurally similar proteins. Smoothing methods that indiscriminately spread mass between bins may not include structural constraints of the protein or capture relationships between bins resulting from biochemically favorable protein configurations. By working in the continuous space of blurry neighborhoods as opposed to the discrete space of *n*-hot vectors, we can more meaningfully compare the local structure between two amino acids.

The blurry neighborhood of an amino acid is the expected distribution over bins that its neighbors would occupy after one “evolutionary timestep.” Our training set of aligned structures contains pairs of proteins in the same family, superfamily, or fold; we do not assume that the aligned pairs are homologous (e.g. they may be similar due to convergent evolution rather than a shared common ancestor). However, for simplicity in computing our blurry neighborhoods we consider each pair aligned structures in our training set to be one “evolutionary time step” apart. Further details of dataset preparation are included in Section 2.6.

We generate blurry neighborhoods with a learned transition matrix that encodes the probabilities of neighbors moving between bins. Thus, the blurry neighborhood intrinsically incorporates information regarding the relatedness of particular bins. The construction of a blurry neighborhood is a multi-step process, which is outlined as follows.

Define matrix $C\in {\left(Z_{\geq 0}\right)}^{\left(B+1)\times (B+1\right)}$ where $C_{ij}$ represents how often a neighbor in one structure is in bin *i* and the analogous neighbor in the aligned structure is in bin *j*. Formally, let $C_{ij}$ be the number of times we observe aligned pairs of positions $x_{a},x_{b}$ in protein *X* and $y_{a},y_{b}$ in protein *Y* (across all training structural pairwise alignments) in which $x_{a}$ is aligned to $y_{a}$, $x_{b}$ is aligned to $y_{b}$, $x_{b}$ is in bin *i* of the discretized sphere around $x_{a}$, and $y_{b}$ is in bin *j* of the discretized sphere around $y_{a}$. If $y_{b}$ is not within 15Å of $y_{b}$, this pair contributes to the count $C_{i,-1},$ where negative one represents an extra bin indicating an amino acid that is not within 15Å. Since it is not meaningful which protein serves as *X* and which as *Y* within each pair, we symmetrize by setting $F=(C+C^{⊺})/2.$

We construct transition matrix $T\in R^{\left(B+1)\times (B+1\right)}$ by assigning $T_{ij}=\frac{F_{ij}}{\sum_{k=1}^{B+1}F_{ik}}.$ Thus, $T_{ij}$ represents the probability of a neighbor moving from bin *i* to bin *j* after one step of evolution. The blurry neighborhood $\vec{b}\in {\left(Z_{\geq 0}\right)}^{B}$ represents the expected distribution of neighbors of an amino acid after an evolutionary timestep. We compute the blurry vector $\vec{b}$ from the *n*-hot vector $\vec{v}$ as follows. We append the value 4 to $\vec{v}$ to represent neighbors in the negative one bin and call this (B+1)-dimensional vector ${\vec{v}}^{\prime }$. The value of 4 is a pseudocount calculated from the empirically observed average number of amino acids outside the 15Å sphere that are aligned to an amino acid within the corresponding 15Å sphere in the structurally similar protein. This accounts for the expected value of neighbors outside the 15Å radius that may move into this radius and yields the nice property that applying the transition matrix does not affect the population-wide average number of neighbors. Then we obtain $\vec{b}$ by computing $\left({\vec{v}}^{\prime }T\right)$ and ignoring the position corresponding to the negative one bin.

### 2.2 Comparing local amino acid neighborhoods with the weighted Jaccard similarity

We utilize the weighted Jaccard similarity to compare the similarity between the blurry neighborhoods corresponding to two amino acids from different proteins. Let $\vec{x}$ and $\vec{y}$ represent the blurry neighborhoods associated with amino acids *x* and *y*, respectively. In this case, the weighted Jaccard similarity is defined as follows


(1) $$J(\vec{x},\vec{y})=\frac{\sum_{k=1}^{B}\min(x_{k},y_{k})}{\sum_{k=1}^{B}\max(x_{k},y_{k})},$$

where $x_{k}$ and $y_{k}$ represent the *k*th element of $\vec{x}$ and $\vec{y},$ respectively. Note that by construction, $0\leq J(\vec{x},\vec{y})\leq 1.$ If blurry neighborhoods $\vec{x}$ and $\vec{y}$ are identical, they will have weighted Jaccard similarity of 1, and if they have 0 overlap in each bin, they will have weighted Jaccard similarity of 0. This is a generalization of the Jaccard index of set theoretic origin, which reflects the similarity between two sets by taking the ratio of their intersection to their union.

### 2.3 Discretizing blurry neighborhood representations into a 3Dn alphabet

We can reduce the computational overhead of the blurry neighborhood-based structure comparison process by clustering neighborhoods into a 3Dn alphabet. This allows us to compare two blurry neighborhoods by looking up a score between their corresponding characters as opposed to computing the weighted Jaccard similarity. To discretize the blurry neighborhoods, we use a graph clustering approach to define 20 “landmark” vectors and associate each with a character. We then assign each amino acid to the character associated with the landmark vector that is nearest with respect to the weighted Jaccard similarity.

**Figure 2.**
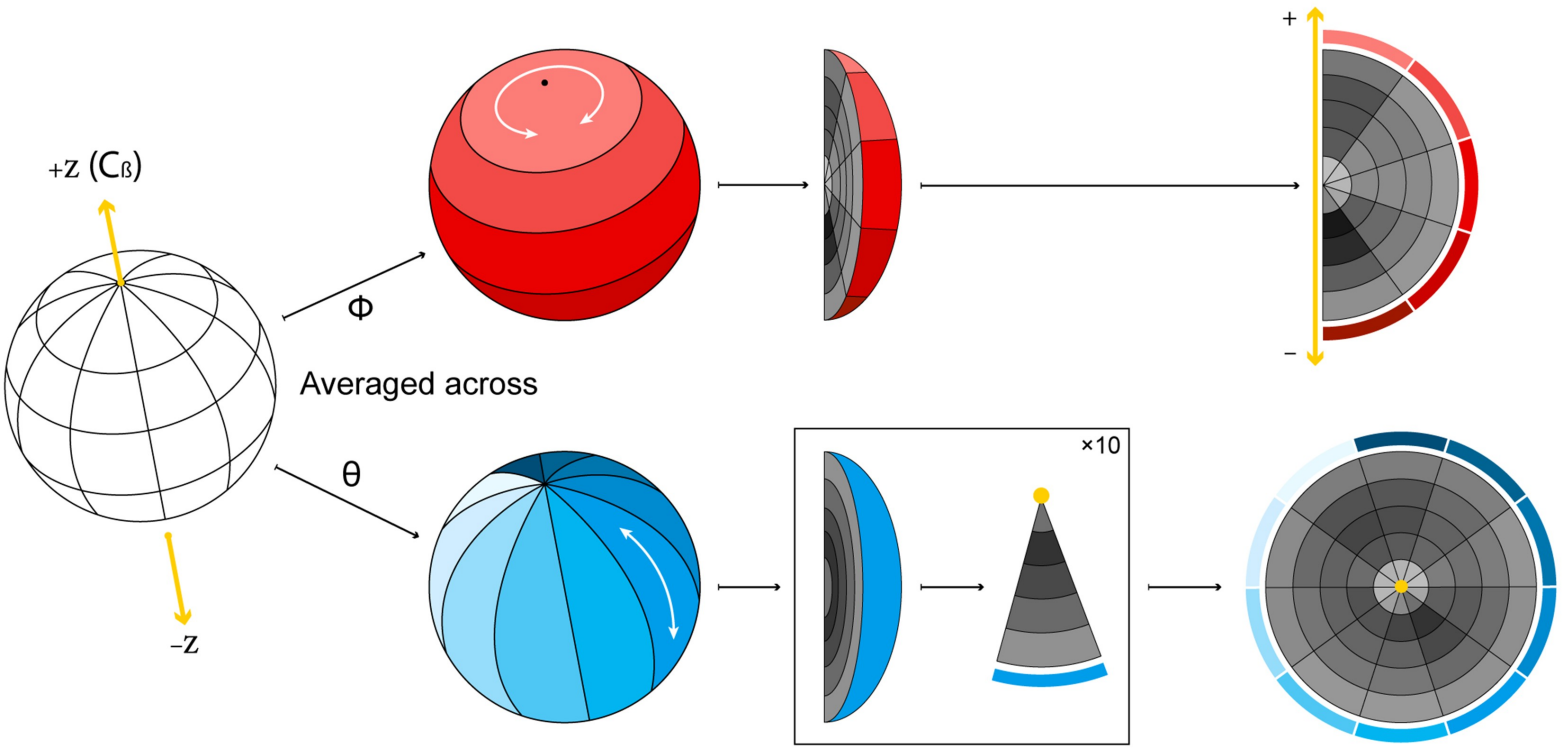
Visualizing 3Dn characters. We discretize the 15 Å sphere oriented with respect to the reference frame depicted in Fig. 1A into 250 bins by taking 10 intervals in ϕ, 5 intervals in θ, and 5 intervals in *r* (hidden in the left image). To visualize the neighbor counts in each of these bins, we show two flattened images obtained by averaging over ϕ (upper row) and θ (lower row). In the upper row, the darkness of each cell represents the average number of neighbors across all bins with a particular interval for *r* and interval for θ. In the lower row, the darkness of each cell represents the average number of neighbors across all bins with a particular interval for *r* and interval for ϕ. The *z*-axis appears as a line in the upper plot and a point in the lower plot, as depicted in yellow. See Fig. 3 for examples. Note that these figures are not to scale; true *r*, θ, and ϕ values are chosen so that each bin has equal volume.

**Figure 3.**
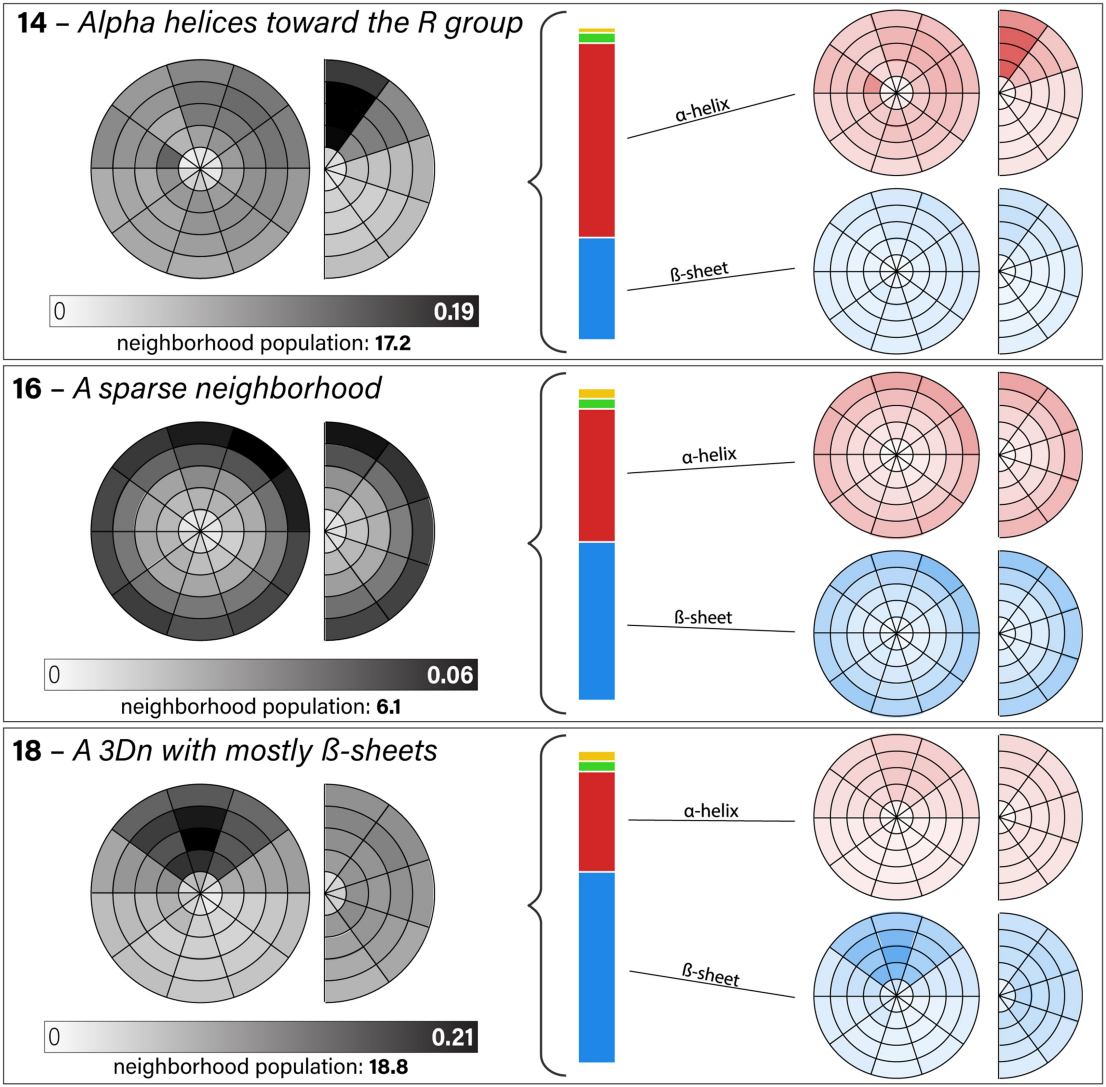
Visualization of 3Dn characters 14, 16, and 18. Figure 2 explains how to interpret the circle and semi-circle figures. The left figures illustrate the spatial distributions of neighbors in the landmark blurry neighborhood for the 3Dn character. The number of neighbors varies substantially across the different 3Dn states, as indicated by the given neighborhood populations. The vertical bar shows the distribution of the secondary structure of the neighbors. The top rightmost plots illustrate the distribution of the right helix neighbors, whereas the bottom rightmost plots illustrate the distribution of beta sheet neighbors. See Section 3.2.1 for a qualitative description of the three 3Dn states visualized here. The full alphabet, including left α-helix (yellow) and unclassified secondary structure (green) is depicted in Fig. 12, available as supplementary data at *Bioinformatics* online.

To construct the landmark blurry neighborhoods we cluster randomly selected 20 000 blurry neighborhoods ($\vec{b}$) of amino acids from different protein structures. We construct a graph by connecting a pair of blurry neighborhoods $\vec{x}$ and $\vec{y}$ by a weighted edge with the edge weight equal to the weighted Jaccard similarity $J(\vec{x},\vec{y})$ between them. To make this graph sparse, we discard the low weight edges and only keep 20 nearest neighbors for each node. Then, we use Louvain community detection algorithm with the resolution parameter tuned to give 20 final clusters that discretize the neighborhoods (Reichardt and Bornholdt 2006, Blondel *et al.* 2008). For each cluster, we define the landmark blurry neighborhood by taking the median entry-wise of the blurry neighborhoods $\vec{b}$ in that cluster. We can visualize the landmark blurry neighborhoods, which allows us to qualitatively view the local structure encoded by each character in the 3Dn alphabet, see Figs 2 and 3.

We considered two other approaches for discretizing our neighborhood representations: (i) training a VQ-VAE on the *n*-hot neighborhood vectors and (ii) further optimizing our graph cluster centers using a mutual information objective. Neither were as successful as the graph clustering approach, see Section A.3.

### 2.4 Constructing an alphabet based on backbone dihedral angles

As a point of comparison, we construct an alphabet derived only from the backbone dihedral angles. First, we discretize the space of ϕ and ψ backbone dihedral angles with a 30 by 30 grid, resulting in 900 bins. Many of these bins are in infeasible regions of Ramachandran plot or only account for a very small number of examples, so we reduce the number of bins to 251. Using a mutual information objective, we cluster the bins to obtain a dihedral alphabet with 20 characters. We consider the distribution over pairs of bins given by the empirical frequency of how often we observe each pair of bins substituting for each other among aligned positions in our training set of aligned structures. Our clustering algorithm iteratively merges the pair of adjacent bins that results in the distribution with maximal mutual information. See Appendix A.5, available as supplementary data at *Bioinformatics* online for details. Figure 6, available as supplementary data at *Bioinformatics* online depicts the regions of Ramachandran plot corresponding to each character and the corresponding learned dihedral substitution matrix.

### 2.5 Computing alignments

In order to determine an alignment between two proteins using the Smith-Waterman local sequence alignment algorithm (Smith and Waterman 1981), it is first necessary to construct a suitable similarity matrix *M*, where $M_{ij}$ quantifies the structural similarity between the *i*th amino acid in the first protein and the *j*th amino acid in the second. In our blurry neighborhood method, $M_{ij}$ is determined by a log-odds transformation of the weighted Jaccard similarity applied to the corresponding blurry neighborhoods (see Appendix A.4, available as supplementary data at *Bioinformatics* online). For our 3Dn alphabet, we compute a substitution matrix on the 3Dn characters (see Appendix A.4, available as supplementary data at *Bioinformatics* online), and let $M_{ij}$ be the score for the corresponding pair of 3Dn characters. When we combine *n* different alphabets, we instead consider matrix $M=\sum_{i=1}^{n}\alpha_{i}M_{i},$ where $M_{i}$ is the contributed matrix using alphabet *i*; the weightings $\alpha_{i}>0$ are determined in a search process outlined in Appendix A.2, available as supplementary data at *Bioinformatics* online. Thus, when we combine two alphabets of size *M* and *N*, we are not constructing a new alphabet of size M×N from the Cartesian product of these alphabets. Instead, we are taking a linear combination of the resulting similarity matrices that occur from independently applying each alphabet to the protein pair of interest.

We construct an alignment between a pair of proteins using the Smith-Waterman algorithm (Smith and Waterman 1981) with the scoring matrix *M* as implemented in Petti *et al.* (2023). We used a hyperparameter search process outlined in Appendix A.2, available as supplementary data at *Bioinformatics* online to determine the gap open and gap extend penalties. After acquiring the local sequence alignment, we evaluate alignment quality using the Local Distance Difference Test (lDDT) (Mariani *et al.* 2013) and use the lDDT to rank the results in our search task. Since our method is a local search method, we use lDDT rather than TM-score (Zhang and Skolnick 2004). While the TM-score measures global similarity, the lDDT measures the extent to which the local structure is conserved, and thus is less sensitive to the movement of protein domains (Mariani *et al.* 2013).

### 2.6 Description of data

For the database search task, we utilize the SCOPe40 dataset (Chandonia *et al.* 2019, Van Kempen *et al.* 2024), a set of 11 211 protein sequences resulting from clustering SCOPe 2.01 at 40% sequence identity. Folds from SCOPe40 were split 80%/20% into training and test sets, with 10% of the training set reserved for validation. The transition matrix and dihedral substitution matrix clusters were trained utilizing a set of TM-alignments of protein pairs with TM-scores of at least 0.6. We used the validation set to train algorithm hyperparameters: the open and extend gap penalty for the Smith-Waterman algorithm and the relative weights of the alphabet combinations. The process detailing the hyperparameter selection process is outlined in Appendix A.2, available as supplementary data at *Bioinformatics* online. When executing the search benchmarking task, we queried each test protein against the entire protein database.

## 3 Results

### 3.1 Search benchmark

We evaluate the efficacy of our blurry neighborhood-based algorithm on a database search task using proteins from SCOPe40 (Chandonia *et al.* 2019), as discussed in Section 2.6. We evaluate the lDDT of the alignment constructed between a fixed query protein and each target protein in the database, and rank the target proteins by descending lDDT value. The efficacy of each query is evaluated using sensitivity up to the first false positive across family, superfamily, and fold levels, which is the ratio of the number of true positives before the first false positive to the total number of true positives at each classification level. We average sensitivities up to the first false positive over all query proteins at each of the family, superfamily, and fold levels to benchmark our algorithm performance at different levels of divergence, see Table 1.

**Table 1.** Comparison of sensitivity up to the first false positive across Family, Superfamily, and Fold categories.^a^

Method	Family	Superfamily	Fold
AA	0.636	0.176	0.006
Dihedral	0.751	0.316	0.035
3Di	0.830	0.431	0.108
3Dn	0.814	0.393	0.085
3Di-AA	0.864	0.491	0.133
3Dn-AA	0.836	0.400	0.069
3Di-Dihedral	0.829	0.450	0.115
3Dn-Dihedral	0.850	0.453	0.103
3Di-3Dn	0.874	0.495	0.151
3Di-3Dn-AA	**0.885**	**0.518**	**0.152**
3Di-3Dn-Dihedral	0.872	0.512	0.150
BV	0.886	0.503	0.121

a AA refers to the amino acid alphabet with BLOSUM62 scoring (Henikoff and Henikoff 1992), Dihedral refers to an alphabet based on backbone dihedral angles described in Section 2.4, 3Di refers Foldseek’s 3Di alphabet (Van Kempen *et al.* 2024), 3Dn refers our proposed alphabet, and BV refers to the scoring method using blurry neighborhood vectors with the Jaccard similarity metric. The relative weights of the combined alphabets as well as the gap penalties for each method are given in Table 2, available as supplementary data at *Bioinformatics* online. The top performing alphabet method is bolded, and the top performing alphabet method that does not require amino acid identity is underlined.

Among the alphabet methods that do not require amino acid identities, the 3Di-3Dn-Dihedral and 3Di-3Dn combination alphabets are the top performers. The overall best performance is achieved by the 3Di-3Dn-AA combination alphabet. Notably, the 3Di-3Dn combination alphabet is a substantial improvement over using each alphabet individually, suggesting that the 3Di and 3Dn alphabets encode different information. We explore these differences in the following section. Moreover, the performance of the 3Di-3Dn and the 3Di-3Dn-Dihedral alphabets are modest improvements over the 3Di-AA alphabet combination used by Foldseek. Thus, we have established a method that does not use amino acid identities but gives comparable results to Foldseek, which is of interest for structure search when homology is not expected.

We also consider the performance of the Blurry Vector (BV) approach, which is an instantiation of our blurry neighborhood method with the discretization utilizing 1000 bins. Our BV method performs similarly to the 3Di-3Dn combination alphabet while also not requiring amino acid identities. However, our BV method is much less efficient than the alphabet methods because the similarity matrix is computed with a weighted Jaccard similarity metric rather than computed via look-ups in a substitution matrix. We include the BV results to draw attention to the discrepancy in performance between the BV method and 3Dn alphabet, suggesting there is a substantial loss in information in the clustering process.

**Figure 4.**
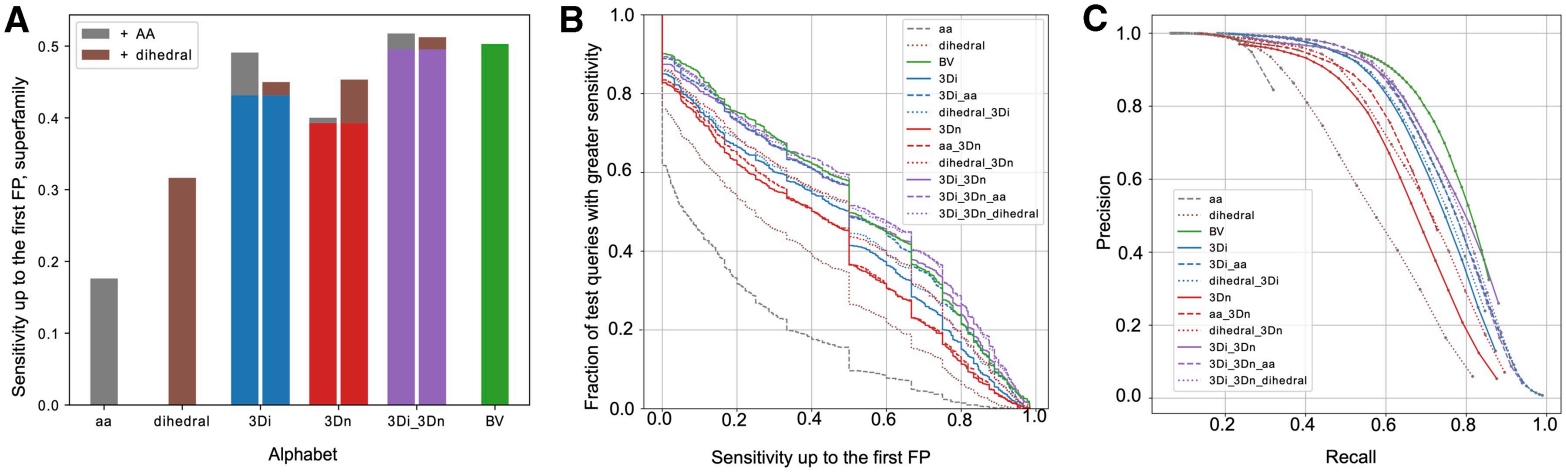
Superfamily search benchmark results. (A) Sensitivity up to the first false positive for AA, Dihedral, 3Di, 3Dn, 3Di-3Dn, and BV alphabets at the superfamily level. The benefit of adding amino acid information is shown in gray, and the benefit of adding dihedral information is shown in brown. (B) The fraction of queries that receive a greater sensitivity up to the first false positive than indicated in the corresponding *x*-axis value, at the superfamily level. Each color and line type represent a different alphabet or combination of alphabets. (C) Precision and recall of each alphabet at the superfamily level. Each color and line type represent a different alphabet or combination of alphabets.

Figure 4A and B illustrates the performance gains achieved by augmenting the 3Di, 3Dn, and combined 3Di-3Dn alphabets with the amino acid and dihedral alphabets, as measured by the average sensitivity up to the first false positive for the Superfamily search. Figure 4C gives the precision recall curves at the superfamily level. Analogous figures for the Family and Fold levels are given in Figs 10 and 11, available as supplementary data at *Bioinformatics* online.

### 3.2 Interpretation of the 3Dn alphabet

#### 3.2.1 Visualization of 3Dn characters

We can visualize each 3Dn character with two figures, constructed by averaging over bins delimited by ϕ (creating a semi-circle) and by θ (creating a circle) as shown in Figure 3. Figure 3 depicts three 3Dn characters: 14, 16, and 18. The 3Dn character 14 is representative of many characters in our alphabet, detecting a particular secondary structure (in this case, right α-helices) toward (or away from) the *R* group. The 3Dn character 16 represents the sparsest neighborhoods with 6.1 neighbors total. The 3Dn character 18 shows β-sheet neighbors uniformly distributed with respect to the angle to the *R* group, but with a distinct pattern with respect to the orientation in our *XY* plane.

#### 3.2.2 Comparison of alphabets: AA, 3Di, dihedral, 3Dn

In order to better understand the biochemical features encoded by Foldseek’s 3Di alphabet and our 3Dn alphabet, we analyze the co-occurrence of amino acid identity, dihedral cluster, and 3Dn and 3Di characters. We find that 3Dn characters encode amino acid identity more than 3Di characters, and 3Di characters encode dihedral information more than 3Dn characters. This finding is consistent with the observation that the 3Dn alphabet exhibits greater improvement when augmented with the dihedral alphabet as compared with the amino acid alphabet, and vice versa for the 3Di alphabet (see Fig. 4A).

**Figure 5.**
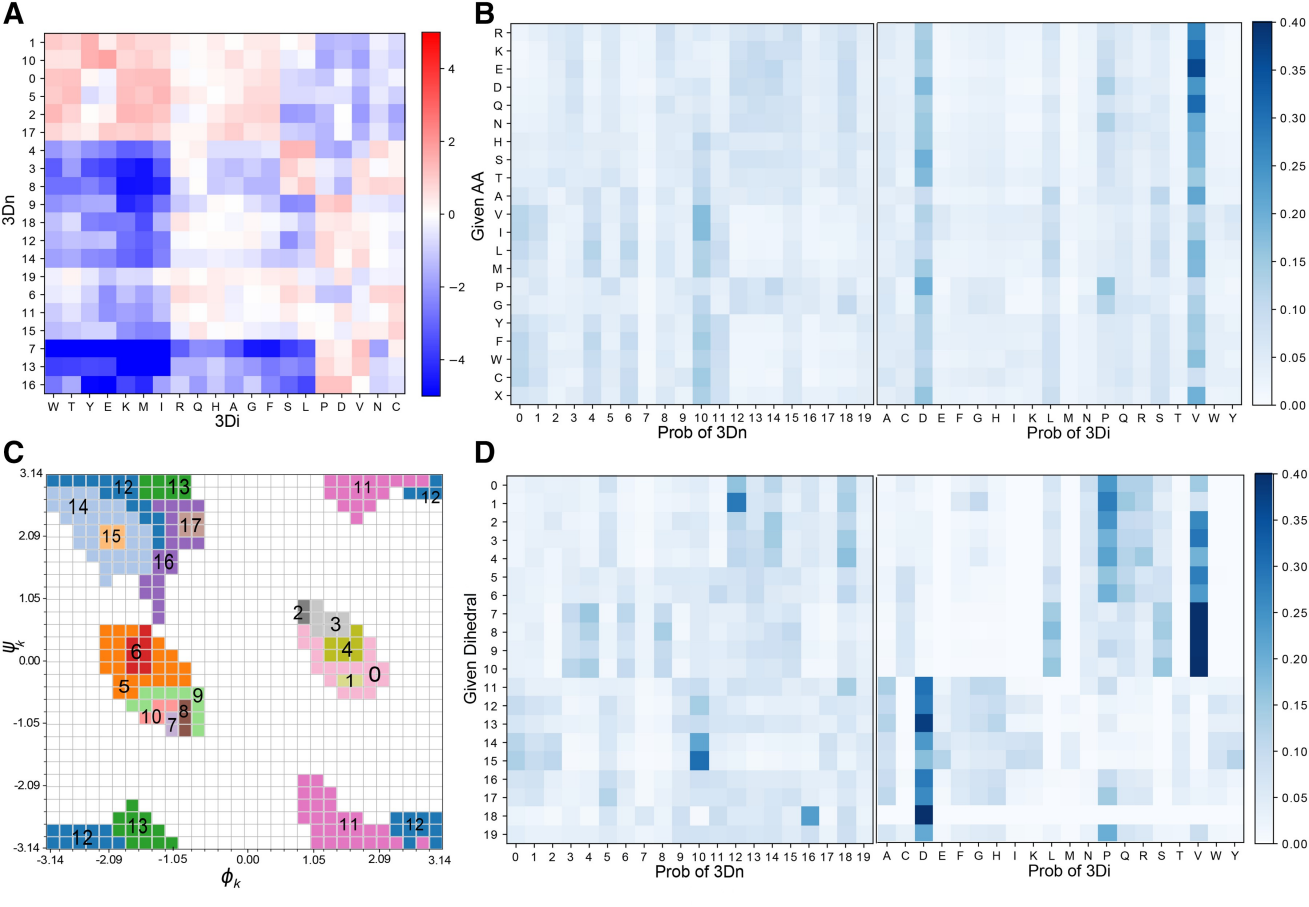
Co-occurance of 3Di, 3Dn, amino acid and dihedral characters. (A) A log odds matrix illustrating the co-occurance of 3Di and 3Dn characters; a positive log odds score (red) indicates the pair co-occur more than what would be expected by chance. (B) Probabilities of any given amino acid belonging to any 3Dn character or 3Di character. (C) Labels of regions corresponding to our learned dihedral characters on a Ramachandran plot. (D) Probabilities of any given dihedral character associating to any given 3Dn character or 3Di character.

First, we explore the relationship between 3Dn and 3Di characters. Recall from Table 1 that combining 3Dn and 3Di alphabets on the search benchmarking tasks outperforms the individual performance of the 3Dn and 3Di alphabets, suggesting that 3Dn and 3Di alphabets capture different structural information. We confirm this intuition by analyzing the co-occurrence of 3Dn and 3Di characters. We compute the ratio between how often each 3Di and 3Dn character pair co-occurs (e.g. how many times we observe an amino acid assigned to the particular pair of 3Di and 3Dn characters) and how often we would expect for them to co-occur under the null distribution that 3Di and 3Dn characters are assigned at random with probability given by their empirical frequencies. Figure 5A, depicts the log of these ratios; the dark blue regions indicate pairs that rarely co-occur, and the red indicates pairs that co-occur more than would be expected by chance. We do not observe a clear mapping between 3Di and 3Dn characters.

Next we compare the co-occurrence of 3Dn and 3Di characters to amino acid identity to evaluate the extent to which each alphabet encodes amino acid identity (Fig. 5B). We observe that certain 3Dn characters tend to exclusively represent polar amino acids or exclusively represent nonpolar amino acids. For instance, 3Dn characters 12, 13, and 14 are more common among polar amino acids, and 3Dn characters 4 and 6 are more common among nonpolar amino acids. Such patterns are not as prominent among the 3Di alphabet, perhaps explaining why when amino acid information is added, 3Di search performance is substantially boosted whereas 3Dn search performance only marginally improves (see gray bars in Fig. 4A).

Similarly, Fig. 5D illustrates the extent to which the 3Di and 3Dn alphabets encode secondary structure, as measured by backbone dihedral angles. Figure 5C depicts our dihedral alphabet; the discretization and subsequent clustering process based on mutual information is detailed in Section 2.4 and Appendix A.5, available as supplementary data at *Bioinformatics* online. We observe that the backbone dihedral angles and consequently, secondary structure is reflected in 3Dn characters as well, with 3Dn character 10 common among beta sheets, characters 3 and 4 common among right helices, and character 12 common among left helices. Recall from Fig. 4A that including dihedral information provides a larger performance enhancement for 3Dn characters than 3Di characters in the search benchmark. This may be because 3Di characters appear to already capture more dihedral information than 3Dn characters, indicated by stronger colored regions in the corresponding graph in Fig. 5D. Therefore, including dihedral information with 3Di characters may be redundant in terms of information captured, accounting for the smaller improvement in combining 3Di and dihedral alphabets when compared to combining dihedral and 3Dn alphabets.

Interestingly, the matrices in Fig. 5D differentiate regions in the Ramachandran plot in a way that agrees with the patterns of conservation encoded in the learned dihedral substitution matrix. Although dihedral characters 5, 6, 7, 8, 9, and 10 all correspond to adjacent regions on the Ramachandran plot we witness that characters 5 and 6 have strikingly different relationships to the 3Dn characters than characters 7, 8, 9, and 10, with a similar yet weaker trend also holding for 3Di characters. This suggests that amino acids with dihedral angles in region 5 and 6 form different structural relationships than those in 7–10. This is supported by the dihedral substitution matrix which illustrates that the substitution pattern for characters 5 and 6 differ from that of 7–10, see Fig. 6, available as supplementary data at *Bioinformatics* online.

#### 3.2.3 Comparison of performance by protein characteristics

To further gain insight on the limitations of 3Di and 3Dn alphabets on structural comparison tasks, we investigated whether they perform differently depending on the characteristics of the proteins being aligned. In Fig. 14, available as supplementary data at *Bioinformatics* online, we consider the performance of 3Di and 3Dn methods on query proteins depending on their protein class, a level of protein classification weaker than fold. Here, we find that at family, superfamily, and fold levels, 3Dn performs better with smaller proteins, and at the family level, the 3Dn alphabet performs better with alpha helix dominant proteins. Alternatively, we see better performance at the family and superfamily level by the 3Di alphabet on beta sheet dominant proteins. While further investigation is necessary to fully explain these discrepancies we offer some hypotheses. The 3Dn alphabet encodes structural information on a larger scale spatially than 3Di; 3Dn characters capture all amino acids that are >5 positions away in the sequence and with 15Å. Since alpha helices are more dense than beta sheets, comparatively more helix information may be encoded in the neighborhood. For small proteins, many amino acids are on the boundary of the protein and will have 15Å neighborhoods with large unoccupied regions, which can be expressed by 3Dn characters.

We also consider the performance of 3Di and 3Dn alphabets by considering the alignment quality of pairs. Figure 15, available as supplementary data at *Bioinformatics* online shows the distribution of lDDTs for pairs of various protein classes. We find again that 3Dn performs better on smaller proteins. Additionally, we considered alignment quality by relative protein length, which we defined as $\frac{\min(l_{a},l_{b})}{\max(l_{a},l_{b})},$ where $l_{a}$ and $l_{b}$ refer to the sequence lengths of proteins *a* and *b*, respectively. Here, we do not see any clear performance trends between 3Di and 3Dn alphabets on pair alignments as a function of the relative protein lengths.

In Appendix A.6, available as supplementary data at *Bioinformatics* online, we explore examples of three pairs of proteins where the 3Di-3Dn combined alphabet outperforms both the 3Di and 3Dn alphabets on the alignment task. We find that in two out of the three pairs of proteins, the alignment generated by the 3Di-3Dn alphabet contained regions from both the 3Di alignment and 3Dn alignment, suggesting that the 3Di-3Dn alphabet finds better alignments by selecting high quality regions from each of the 3Di and 3Dn alignments. In the third example, the combination alphabet aligns regions not aligned in either alphabet individually. In Appendix A.7, available as supplementary data at *Bioinformatics* online, we compare the character distributions in 3Di and 3Dn alphabets across different protein classes. The distribution of 3Dn characters varies widely depending on the dominant secondary structures of the protein, whereas the distribution of 3Di characters does not vary as widely between classes of proteins.

## 4 Discussion

We proposed an interpretable method for characterizing local structure in proteins by considering the spatial distributions of nonadjacent amino acid neighbors. Our blurry neighborhood method and corresponding 3Dn alphabet performed well on protein database search tasks, and when combined with 3Di characters from Foldseek, outperformed both the 3Di and the 3Dn alphabets individually. The combined 3Di-3Dn alphabet is the state of the art alphabet for local search that does not rely on amino acid identity.

The default Foldseek setting is search with a 3Di-AA combined alphabet. Therefore, since 3Di-3Dn is also a combination of two alphabets, the computational cost of alignment and search with the 3Di-3Dn alphabet would be the exact same as using the default mode of Foldseek. Pursuing formal integration with Foldseek introduces a series of engineering challenges. First, a limitation of our 3Dn alphabet is that computing the 3Dn characters requires constructing a 1000D blurry neighborhood. A 3Dn alphabet derived from a coarser discretization of the sphere has the potential to be comparably expressive while computationally more efficient. Second, the search algorithm performance of the combined alphabets may benefit from alternative ways of combining the scores, e.g. taking a geometric mean. Third, further investigation into the ranking criteria is merited. Here we rank by lDDT, but Foldseek ranks by a combination of lDDT, TM-score, and bitscore. Fourth, when considering a protein query for a database search, Foldseek involves prefiltering steps to reduce the size of the target set, which is not currently a feature of our work. It remains to be explored whether a combination alphabet improves prefiltering or whether either individual alphabet will suffice.

More broadly, we hope that our work fuels the exploration of novel alphabets and combinations of alphabets for protein structure search. The demonstration that our 3Di-3Dn alphabet outperforms the 3Di alphabet alone establishes proof of principle that protein structure comparison can be improved with more expressive alphabets. Of particular interest is whether recent methods of tokenizing protein structures designed for deep learning (Gaujac *et al.* 2024, Lu *et al.* 2024, Gao *et al.* 2024) could be repurposed for protein structure comparison. We have developed a series of software tools that allow for easy construction of substitution matrices for arbitrary alphabets, a mutual information-based clustering algorithm to reduce size of alphabets, and a benchmark for assessing performance on a search task, all of which can be applied to formulate and test other alphabets.

## Supplementary Material

btaf458_Supplementary_Data

## Data Availability

The data underyling this article are available in https://github.com/spetti/structure_comparison and at Zenodo https://doi.org/10.5281/zenodo.15734371. The datasets were derived from sources in the public domain, particularly SCOPe https://scop.berkeley.edu/.

## Footnotes

1 Inferred beta carbon in the case of glycine.
