# Supplementary material for: An interpretable alphabet for local protein structure search based on amino acid neighborhoods
Source: Bioinformatics. 2025 Aug 23;41(10):btaf458. doi: 10.1093/bioinformatics/btaf458 (PMC12516309; doi:10.1093/bioinformatics/btaf458)
Supplement: btaf458_Supplementary_Data [file btaf458_supplementary_data.pdf]

A. Extended Methods

A.1. Neighborhood construction

We encode spatial location of the neighbors of x by discretizing the 15Å sphere oriented with respect to the reference frame centered at the alpha carbon of x. We create "bins" of equal volume characterized by ranges of R, φ, and θ values, the parameters of the spherical coordinate system. We partition $[0, R_{\text{MAX}}], [0, 2\pi]$, and $[0, \pi]$ into N_R, N_φ, and N_θ subintervals. We define the ith subinterval, 0 indexed, of $[0, R_{\text{MAX}}]$ as $R_i = [(\frac{V}{N_R}\frac{3i}{4\pi})^{1/3}, (\frac{V}{N_R}\frac{3(i+1)}{4\pi})^{1/3}]$, where $V = \frac{4\pi}{3}R^3_{\text{MAX}}$. We define the ith interval of φ as $\varphi_i = [\frac{2\pi i}{N_\varphi}, \frac{2\pi(i+1)}{N_\varphi}]$. We define the ith interval of $[0, \pi]$ as $\theta_i = [\cos^{-1}(1 - \frac{2i}{N_\theta}), \cos^{-1}(1 - \frac{2(i+1)}{N_\theta})]$. For the specific blurry vector implementation in the main text, we partition the 15Å sphere into 250 bins of equal volume, where we partition [0, 15Å], $[0, 2\pi]$, and $[0, \pi]$ into $N_R = 5$, $N_\varphi = 10$, and $N_\theta = 5$ subintervals, respectively.

To encode secondary structure assignment, we utilize set $S = \{\beta, \alpha_R, \alpha_L, X\}$ to represent beta sheets, right alpha helices, left alpha helices, and an unknown secondary structure X, respectively. We assign an element of the secondary structure based on the φ and ψ angles of the corresponding neighbor, with β assigned if $\varphi \in [-3.14, -0.42], \psi \in [0.63, 3.14]$ or $\varphi \in [-3.14, -0.84], \psi \in [-3.14, -2.09]$, α_R assigned if $\varphi \in [-2.51, -0.42], \psi \in [-1.47, 0.63]$, α_L assigned if $\varphi \in [0.63, 2.51], \psi \in [-0.84, 1.26]$, and X assigned otherwise. Our assignments are imperfect since backbone dihedral angles are not a perfect proxy for secondary structure. For instance, amino acids in turns may be assigned to helices, and the first and last amino acid will always be assigned to the extraneous X bin.

In our blurry neighborhood vectors, each position or "bin" corresponds to the Cartesian product of the three intervals in conjunction with the secondary structure set: $R_i \times \varphi_j \times \theta_k \times S$. Once an amino acid y is determined to be a neighbor of reference amino acid x, y will be assigned a corresponding bin. This bin reflects both the location of y relative to x and the secondary structure information of y.

A.2. Validation task to choose weightings and gap penalties

We followed a two step procedure to find the best gap open and extend penalties for each alphabet. To do so, we first divided our validation set into two parts. We identified 93 proteins in the validation set that satisfy the hierarchical condition for search (the data contains at least one member in the same superfamily that is not in the family and at least one member in the same fold that is not in the same superfamily). We call this set the "validation query set." To arrive at the "validation alignment set" we considered all TM-aligned pairs of validation proteins in which both proteins had length less than 512 and neither protein is part of the validation query set. We determined the optimal gap-open and extend as follows:

1. We align all pairs in the validation alignment set with all combinations of gap open parameters $\{-20, -18, \cdots - 2, 0\}$ and gap extend parameters $\{-3, -2.5, -2, \ldots 0\}$. We compute the gap open and extend parameters that give us the best mean lDDT of the resulting alignments and the best spearman correlation between the lDDT of our alignments and the lDDT of the TM-alignments. These parameters tended to differ from each other, and it is not clear apriori which is a better metric to use. For the validation search task, we considered all pairs of open and extend parameters that fell between these two different notions of optimal open and extend (e.g. if the optimal via mean was (a, b) and the optimal via spearman correlation was (c, d) with $a < c$ and $b < d$, we considered any open-extend pair (x, y) from the original list with $a \leq x \leq c$ and $b \leq y \leq d$).
2. We performed a search benchmark for the validation query set against all training and validation proteins for each combination of open and extend in the reduced list determined by the previous task. We computed the average sensitivity up to the first false positive at the family, superfamily, and fold levels. We then selected the gap open and extend parameters with the highest result at the superfamily level.

We followed a similar procedure for determining the optimal weighting when combining different alphabets. In the two alphabet case, we considered weights on the first alphabet of $\{0.3, 0.4, 0.5, 0.6, 0.7\}$ and chose the second alphabet weight so the weights summed to one. In the three alphabet case we considered 15 weighting schemes in which each alphabet had weight at least 0.2 and all weights are of the form $x/10$ for an integer x. We performed the validation alignment task with all combinations of weights and gap open and extend parameters listed above. When the optimal parameters determined by mean lDDT and spearman correlation included the same weighting, we fixed this weighting and followed the procedure above to select the smaller list of gap open and extend parameters for the validation search benchmark. In cases where different weightings produced the best mean lDDT and spearman correlation, we considered all combinations of weightings and gap parameters that yielded mean lDDT or spearman correlations that were sufficiently close to the maximums.

Table A.2 gives the parameters used for each method.

Method	Gap Open	Gap Extend	Weights
AA	-6.0	-0.5	N/A
Dihedral	-6.0	-0.5	N/A
3Di*	-10.0	-1.0	N/A
3Dn	-10.0	-0.5	N/A
BV	-8.0	-1.0	N/A
3Di-AA*	-10.0	-1.0	1.4/2.1
3Dn-AA	-6.0	-0.5	0.4/0.6
3Di-Dihedral	-6.0	-0.5	0.6/0.4
3Dn-Dihedral	-2.0	-0.5	0.3/0.7
3Di-3Dn	-10.0	-0.5	0.6/0.4
3Di-3Dn-AA	-2.0	-0.5	0.4/0.2/0.4
3Di-3Dn-Dihedral	-4.0	-0.5	0.4/0.3/0.3

Table 2. Gap open, gap extend, and weights used for each method. Note that for 3Di and 3Di-AA we used the default parameters given by Foldseek.

A.3. Other discretization approaches

A.3.1. VQ-VAE

Following the success of Foldseek in using a VQ-VAE as an effective clustering tool, we adapted the technique to our n-hot data as follows. The *encoder* maps each n-hot vector through three fully-connected layers to a continuous 2-dimensional latent

representation. This latent representation mapped via the *vector quantizer* to the nearest (ℓ_2) centroid in a learned set of 20 centroids. The *decoder* maps the centroid associated with each input x to a length-1000 vector $\hat{y}$ intended to predict n-hot vector of the amino acid aligned with x. We train the VQ-VAE on pairs of aligned positions using a Jaccard loss function (see Equation (1)).

We associate each of the 20 centroids with a letter in our alphabet. Given a $L \times 1000$ representation of a protein, we render a sequence of length L by running each length-1000 n-hot vector through the encoder and vector quantizer portions of the VQ-VAE. Each encoded, quantized input takes the form of a centroid, associated with some character in our 20-letter alphabet. We assign this character to the position. Note the decoder is used only in training.

Our VQ-VAE was implemented in PyTorch (version 2.4.1) over a custom Jaccard (IoU) loss function, with commitment cost = 0.25, the Adam optimizer, a batch size of 512, and a learning rate of 10^{-3} over six epochs. The encoder portion comprises layers of 1000, 1000, and 20 nodes; the decoder comprises layers of 20, 1000, and 1000 nodes.

Clusters found by VQ-VAE did not reach the quality of the clusters generated by our graph clustering method nor are they as interpretable. The sensitivities up to the first false positive at the family, superfamily, and fold levels were 0.756, 0.333, 0.053 respectively. This is similar to the performance of the dihedral alphabet at the family level and a slight improvement for the superfamily and fold levels.

A.3.2. Optimizing learned centers with mutual information

Our graph clustering approach learns 20 cluster centers and classifies each amino acid as the character corresponding to the closest cluster center with respect to the weighted Jaccard similarity metric. We attempted to further optimize the location of the cluster centers using gradient descent with an objective function that sought to jointly maximize the mutual information and entropy of a substitution matrix computed for random subsets of the training data. After adjusting the cluster centers in this manner, the corresponding alphabet yielded sensitivities up to the first false positive at the family, superfamily, and fold levels of 0.804, 0.385, 0.077 respectively. This performance was marginally worse than our graph-cluster derived 3Dn alphabet.

A.4. Details on scoring schemes

The following details the computation of the entries M_{ij} of similarity matrix between two proteins with the blurry neighborhood method. First we compute the weighted Jaccard similarity between the blurry neighborhoods of the ith amino acid in the first protein and the jth amino acid in the second protein. Then we apply a transformation that maps the value of the weighted Jaccard similarity to a log-odds score. We compute this transformation as follows. We compute the weighted Jaccard similarity for pairs of aligned positions and non-aligned positions in the the training set. We bin the Jaccard values into 100 bins (e.g. 0-0.01; 0.01-0.02; ...0.99-1.0). For each bin, we take twice the *log* of the ratio of the number of aligned pairs with Jaccard value in that bin to the total number of pairs with Jaccard value in that bin. This value is the transformed weighted Jaccard similarity score for the bin. We manually adjust the values for high and low Jaccard bins with little data.

For our alphabets, the entry M_{ij} is given by the score in the substitution matrix for the ith character of the first sequence and the jth character of the second sequence. Our substitution matrix scores are computed as follows:

$$B_{kl} = \log_2 \left(\frac{f_{kl}}{f_k f_l} \right),$$

where f_{kl} is the the observed frequency of alignment of characters k and l, and f_k and f_l are the observed frequency of characters k and l, respectively.

A.5. Dihedral substitution matrix

Here we describe the procedure to construct an alphabet using dihedral angles. We start by computing the backbone dihedral angles ϕ and ψ for proteins in our dataset. The distribution of these angles (points on the Ramachandran plot) is binned into 30×30 bins (bins of size $12° \times 12°$) to identify high-frequency bins that each account for at least a threshold fraction ($> 1/900$) of total number of points. We use the same threshold separately for PRO and GLY due to their propensity to occupy non-standard positions in the Ramachandran plot. Points that are not in these high-frequency bins are discarded and we only focus on these high-frequency bins for downstream analyses. Thus, the procedure reduces the total number of bins from 900 to 251.

Next, we construct a 251×251 substitution probability matrix P that expresses how often two bins substitute for each other among structurally aligned protein pairs. An element in the matrix is given by $p_{ij} = f_{i,j} / \sum_{k=1}^{s} f_{i,k}$, where $f_{i,j}$ is the number of times bin i substitutes for bin j and s is the alphabet size. The denominator counts the total number of times bin i substitutes for any bin.

Then we reduce the number of bins by performing a clustering algorithm on the probability matrix that seeks to merge bins in a way that maximizes the mutual information of the distribution. The mutual information of a probability matrix P is $MI = \sum_{i,j} p_{ij} \log(p_{ij})/(p_i p_j)$. We merge a pair of bins results in the maximum MI over the new set of $n-1$ bins. The idea is to have a small number of bins without significant decrease in MI. When attempting to merge a pair of bins, we only consider the bins that are adjacent with periodic boundary condition. We continue merging bins until no further bins can be merged, i.e. no remaining bin pairs are contiguous. Our goal is to construct a 20-character alphabet for a fair comparison with other methods. We select 18 as the appropriate number of hyperbins (clusters of original 251 bins, labeled 0 - 17). Two additional characters are included in the alphabet: one for endpoints of the sequences where the dihedral angles cannot be computed (18), and the other for angles that fall outside the high-frequency bins (19). The structure of a protein can thus be represented as a sequence of these dihedral cluster labels (similar to amino acid sequence), which we use to construct the dihedral substitution matrix.

A.6. Case Studies

To gain insight into increased performance of the 3Di-3Dn alphabet, we consider specific pairs of proteins for which the lDDT of the alignment generated by using the 3Di-3Dn combined alphabet is substantially higher than the lDDT of both the 3Di and 3Dn alignments. In Figure 7A, we illustrate the difference in lDDT of alignments generated with the 3Di-3Dn combined alphabet in comparison to the lDDT of the alignments generated

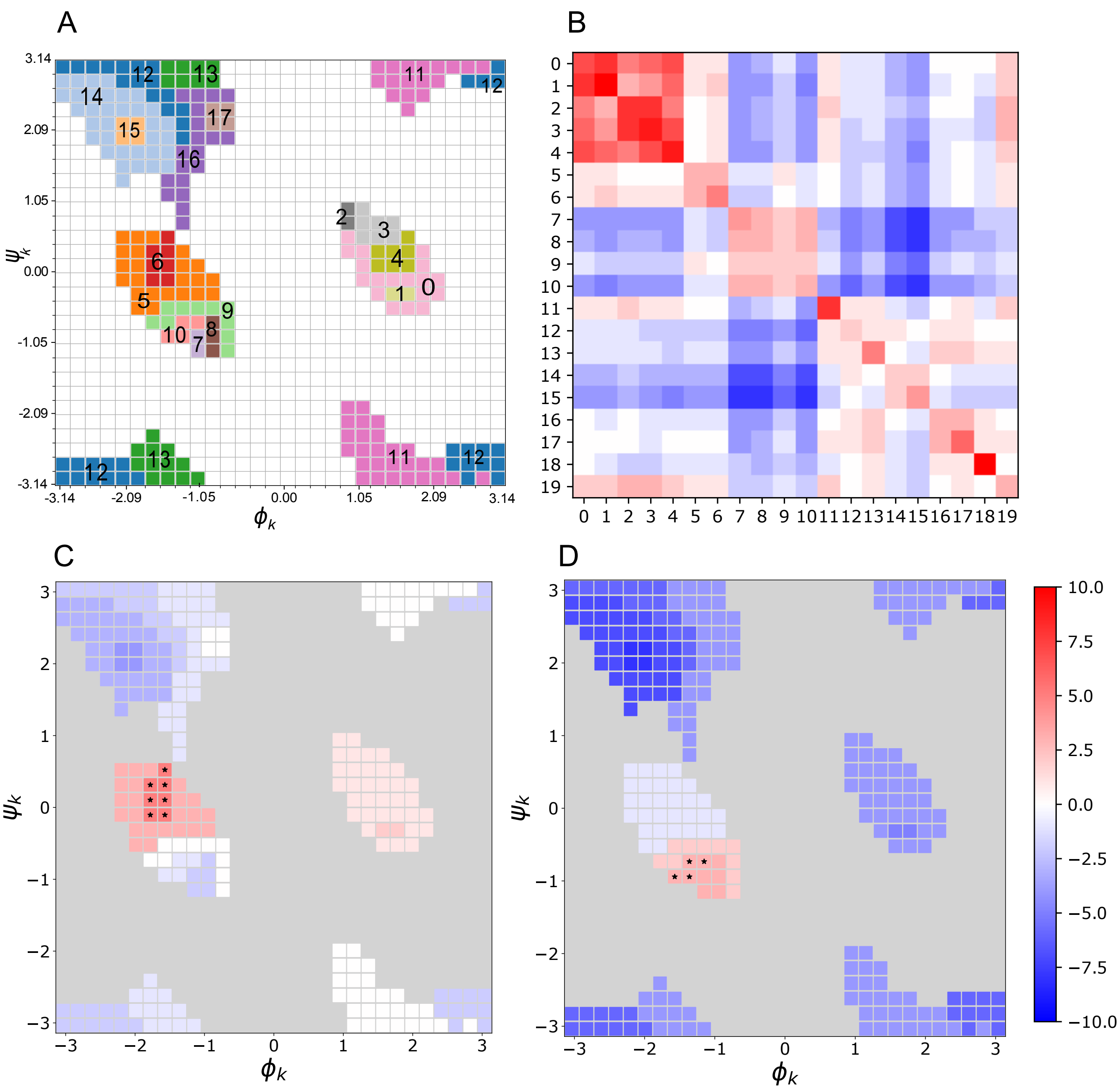

Fig. 6: **Backbone dihedral alphabet and corresponding substitution matrix.** A. Labeled clusters on a Ramachandran plot. B. BLOSUM-like substitution matrix for dihedral alphabet. C and D. Substitution scores between a chosen cluster i (indicated by a set of stars) and all other clusters visualized on a Ramachandran plot, with $i = 6$ in C and $i = 10$ in D.

with the 3Di or 3Dn alphabets individually. The majority of points lie in the upper right quadrant, indicating that for most pairs of structurally similar proteins, the 3Di-3Dn combined alphabet outperforms both the 3Di and 3Dn alphabets individually. We select three protein pairs, colored in green, red, and purple, such that the alignment generated by the 3Di-3Dn combined alphabet has an lDDT larger than each of the 3Di and 3Dn alignments by at least 0.2.

In Figure 7B, C, and D, we visualize the alignments for three such pairs. The fully saturated colors represent the 3Di-3Dn alignment, with grey indicating a positions that were also aligned by both 3Di and 3Dn individually, red indicating positions that were also aligned by 3Dn but not 3Di, blue indicating positions that were also aligned by 3Di but not 3Dn, and purple indicating positions that were not aligned by 3Dn or 3Di individually. For reference we include the other positions aligned by 3Dn and 3Di individually in light red and blue respectively. Observe that in Figure 7C and Figure 7D, the 3Di-3Dn alignment includes regions that were aligned in either the 3Di or 3Dn alignments (see dark blue and dark red regions, respectively), suggesting

that 3Di-3Dn alphabet produced a better alignment by finding the highest quality parts of each alignment. In contrast, the 3Di-3Dn alignment depicted in Figure 7B includes some components of the 3Dn alignment (see dark red region), yet most of the 3Di-3Dn alignment does not overlap with the alignment of either alphabet (see purple region). Thus, the advantages in alignment quality of combining the 3Di and 3Dn alphabets extend beyond the individually contributed regions from each of the 3Di and 3Dn alignments. The purple represents regions that are not sufficiently high scoring under each alphabet individually to be aligned by Smith-Waterman, but whose scores under the combination alphabet become relatively higher (as compared to other regions in the combination scoring matrix), leading to their inclusion in the alignment.

A.7. Extended comparison of alphabets

We considered the character distributions of the 3Di and 3Dn alphabets across various classes of proteins. In Figure 9, we study the distribution of characters used to encode proteins in the SCOPe40 dataset, organizing by protein class. We arrange the 3Dn characters in descending order of the right helix percentage of their associated landmark blurry neighborhoods. A detailed image of the secondary structure composition of each landmark blurry neighborhood is available in Figure 8. Here, we observe that each class of proteins has a distinct pattern of 3Dn character usage. Furthermore, the secondary structure composition of the landmark blurry neighborhoods associated with the most used 3Dn characters within a class reflect the secondary structure of the class. Proteins dominated by alpha helices favor 3Dn characters representing neighborhoods dominated by right helix neighbors (e.g. 4, 8, and 3) whereas proteins dominated by beta sheets favor 3Dn characters representing neighborhoods dominated by beta sheets (e.g. 0, 2, and 5). The a+b class of proteins have alpha helix and beta sheets in separate, contiguous regions, whereas a/b proteins have alternating alpha helix and beta sheet components. Accordingly, we see that proteins in these classes use the full 3Dn alphabet, including the 3Dn characters representing neighborhoods with a significant population of both beta sheets and helices (11, 14, 19, 15, 13, 17, 10, 1, and 6). One notable difference is that the a/b proteins tend to use character 10 more frequently than the a+b proteins. Examining the description of the landmark blurry neighborhood associated with character 10 in Figure 8, we see that 10 is the most populous character and there is no strong spatial pattern of where the neighbors are located. Character 10 may be more common among a/b proteins where the helices and sheets are less separated from each other. Finally, note that the small proteins preferentially use 3Dn characters associated with sparse neighborhoods (3, 7, 16, 9, 12, and 18). Although 3Di has a noticeable shift in character distribution as well across different protein classes, with character “V” heavily in use in protein class alpha and “D” heavily used in protein class beta, the trend is not as pervasive as with 3Dn.

B. Supplemental Figures

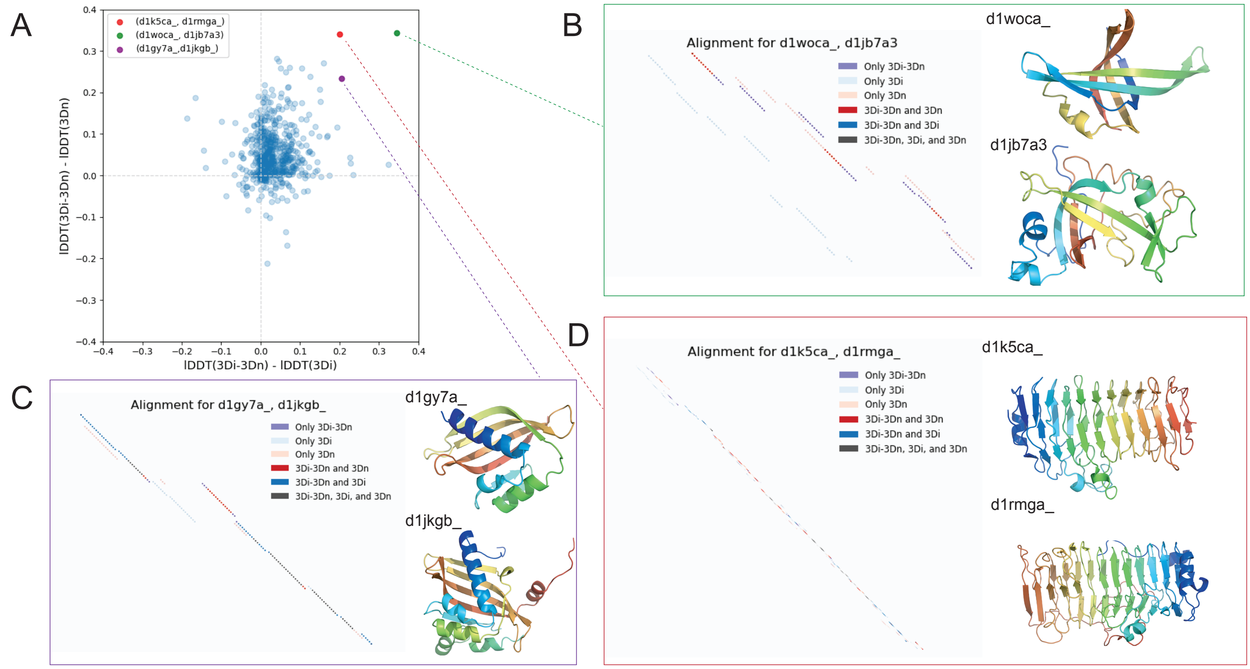

Fig. 7: **Alignment examples using 3Di-3Dn, 3Di, and 3Dn alphabets**. A. Alignments produced by the 3Di-3Dn combined alphabet tend to have higher lDDT than alignments produced by each alphabet individually. Each point represents a protein pair, with the x position as the difference between the lDDT of the 3Di-3Dn based alignment and the lDDT of the 3Di based alignment, and the y position as the difference between the lDDT of the 3Di-3Dn based alignment and the lDDT of the 3Dn based alignment. Three example pairs where the 3Di-3Dn combined alphabet highly outperforms both the 3Di and 3Dn alphabets are highlighted in green, red, and purple. B, C, and D. Alignments for three designated protein pairs. The colored segments indicate the overlap between 3Di-3Dn, 3Di, and 3Dn alignments: gray regions segments show regions where 3Di-3Dn, 3Di, and 3Dn alignments coincide (overlap of all three alignments); dark blue segments denote regions shared exclusively by the 3Di-3Dn and 3Di alignments; dark red segments denote regions shared exclusively by the 3Di-3Dn and 3Dn alignments; purple segments denote regions unique to the 3Di-3Dn alignment; light blue segments denote regions unique to the 3Di alignment; and light red segments denote regions unique to the 3Dn alignment. The right shows SCOPe images of each protein in the pair [Chandonia et al., 2019]. The vertical axis represents sequence positions of the first protein in the pair, and the horizontal axis represents sequence positions of the second protein in the pair. The lDDT values for the three protein pairs under each alignment are as follows: (d1woca_,d1jb7a3): (3Di-3Dn: 0.454, 3Di: 0.109, 3Dn: 0.111), (d1gy7a_,d1jkgb_): (3Di-3Dn: 0.699, 3Di: 0.494, 3Dn: 0.465), (d1k5ca_,d1rmga_): (3Di-3Dn: 0.574, 3Di: 0.374, 3Dn: 0.233).

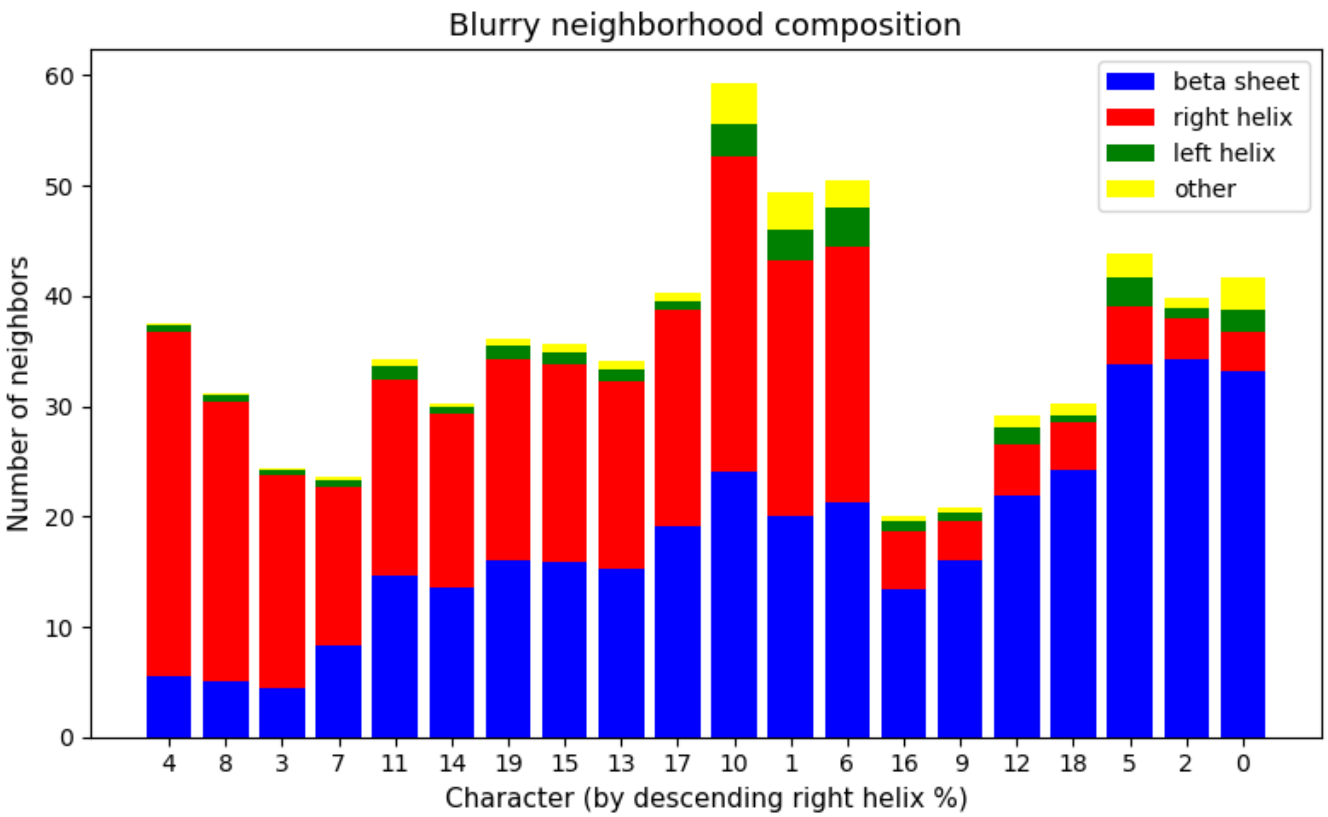

Fig. 8: **Secondary structure composition of landmark blurry neighborhoods**. Demonstrates secondary structure population of the landmark blurry neighborhoods affiliated with each 3Dn character. The x-axis is sorted by descending proportion of right alpha helix composition within the blurry neighborhoods.

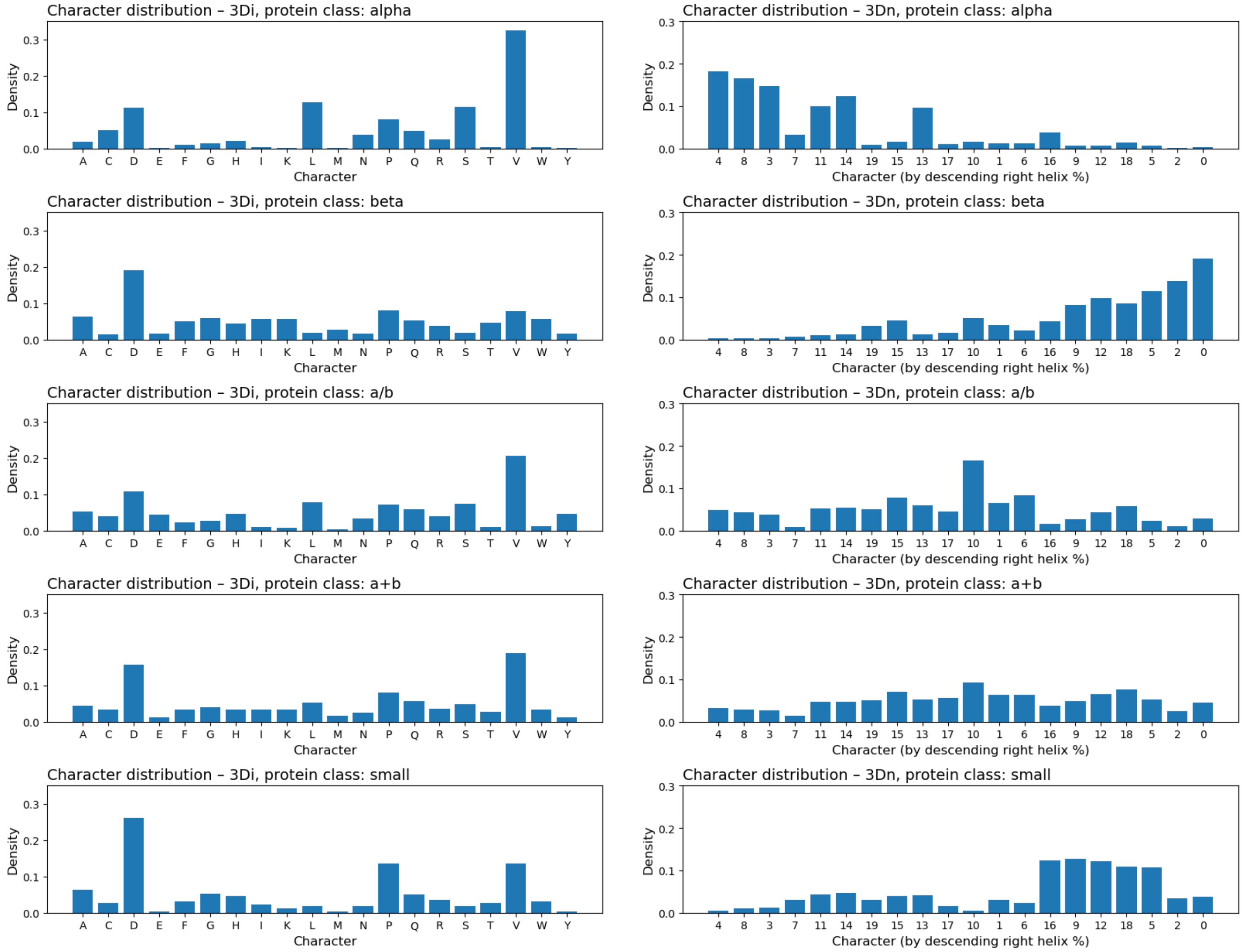

Fig. 9: **Character distributions of 3Di and 3Dn alphabets across different protein classes**. The left set of images depict the distributions of 3Di characters across different protein classes, whereas the right set of images depict the distribution for 3Dn characters across protein classes. The 'alpha' class consists of proteins built almost entirely from alpha helices, the 'beta' class consists of proteins consisting almost entirely of beta sheets, the 'a/b' class refers to proteins with alternating regions of alpha helix and beta sheet components, the 'a+b' class refers to proteins where alpha helix and beta sheet components exist in separate, contiguous regions in the protein, and the 'small' class refers to small proteins. In the right image, the 3Dn characters are organized in order of descending right helix percentage population, as derived from analyzing the population of the corresponding landmark blurry neighborhoods of each character.

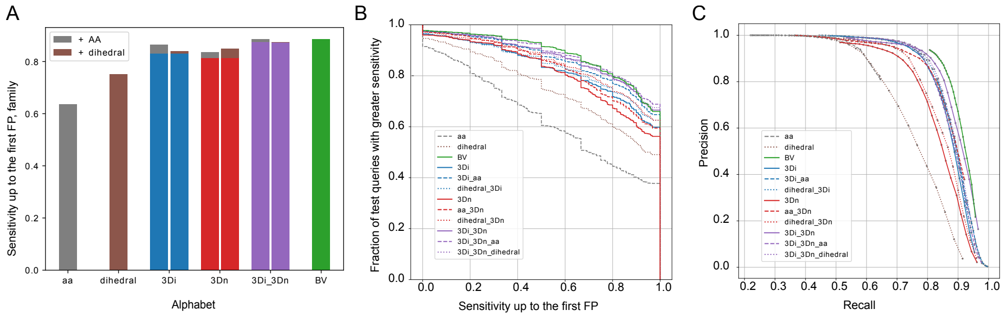

Fig. 10: **Family search benchmark results.** A. Sensitivity up to the first false positive for AA, Dihedral, 3Di, 3Dn, 3Di-3Dn, and BV alphabets at the family level. The benefit of adding amino acid information is shown in gray, and the benefit of adding dihedral information is shown in brown. B. The fraction of queries that receive a greater sensitivity up to the first false positive than indicated in the corresponding x-axis value, at the family level. Each color and line type represent a different alphabet or combination of alphabets. C. Precision and recall of each alphabet at the family level. Each color and line type represent a different alphabet or combination of alphabets.

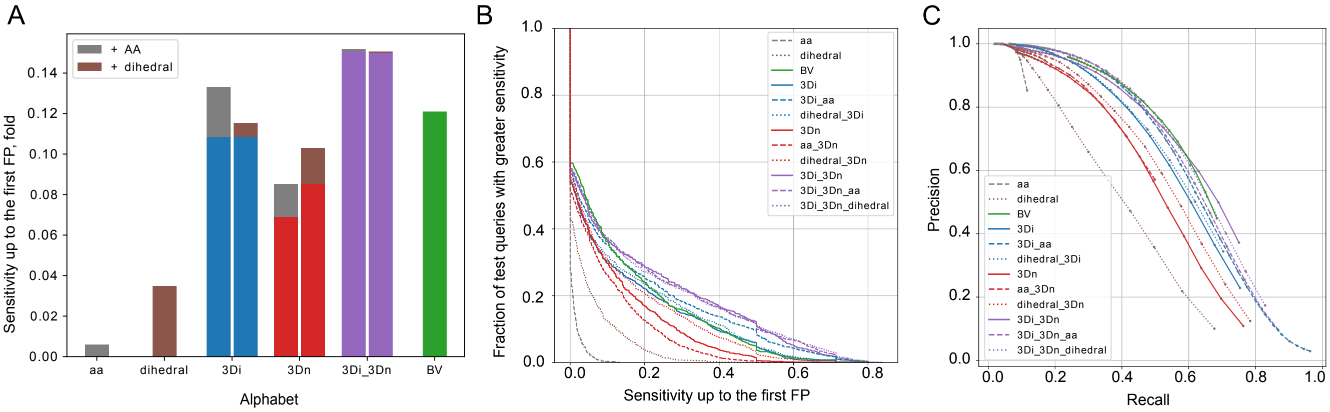

Fig. 11: **Fold search benchmark results.** A. Sensitivity up to the first false positive for AA, Dihedral, 3Di, 3Dn, 3Di-3Dn, and BV alphabets at the fold level. The benefit of adding amino acid information is shown in gray, and the benefit of adding dihedral information is shown in brown. B. The fraction of queries that receive a greater sensitivity up to the first false positive than indicated in the corresponding x-axis value, at the fold level. Each color and line type represent a different alphabet or combination of alphabets. C. Precision and recall of each alphabet at the fold level. Each color and line type represent a different alphabet or combination of alphabets.

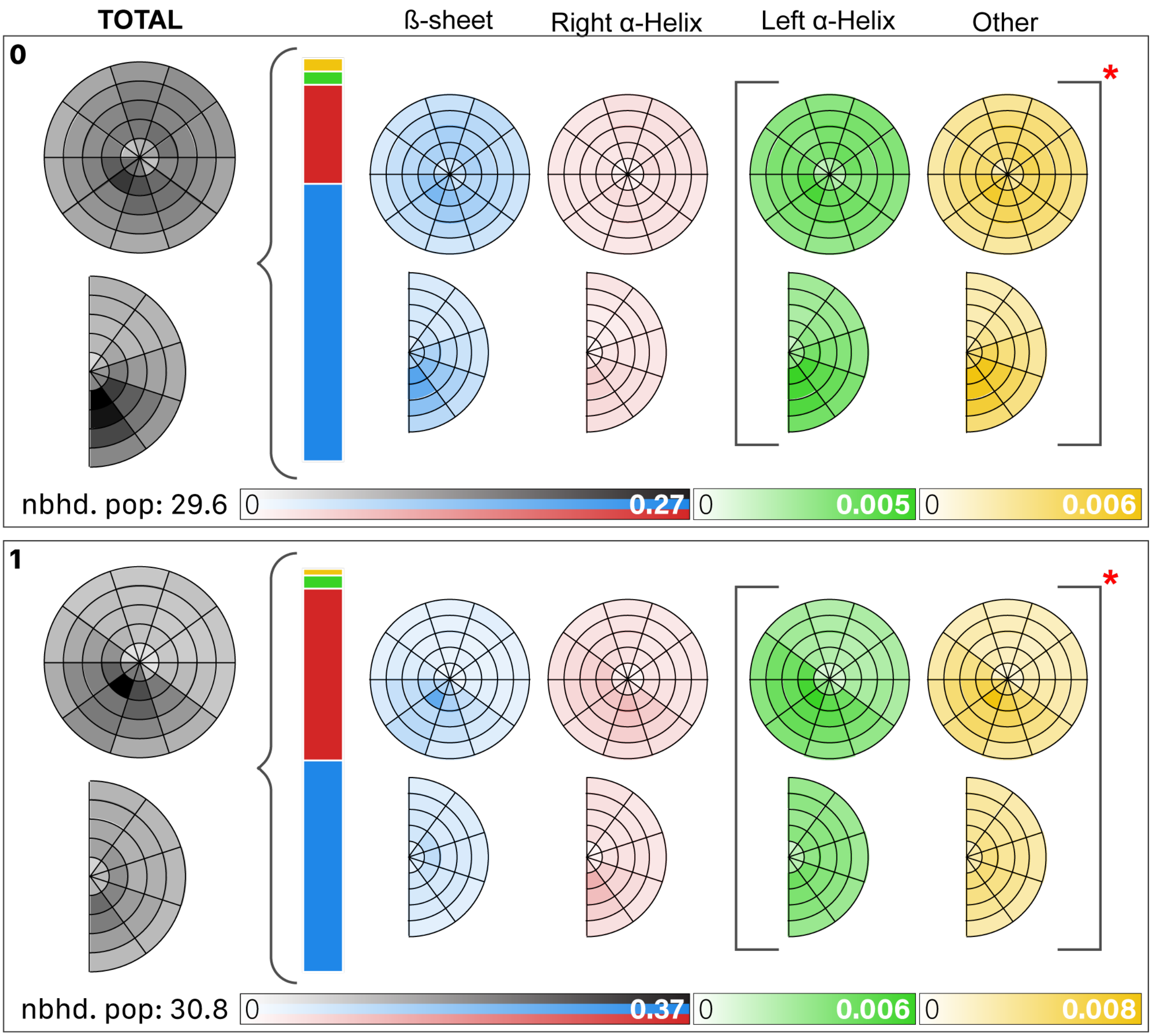

Fig. 12: **The complete 3Dn alphabet.** Figure 3 explains how to interpret the circle and semi-circle figures. The left gray figures illustrate the spatial distributions of neighbors in the landmark blurry neighborhood for the 3Dn character. The number of neighbors varies substantially across the different 3Dn states, as indicated by the given neighborhood populations and different scales of the black, blue, and red colorbar between different 3Dn characters. The four pairs of circles and semi-circles depict the distribution of neighbors by secondary structure type; from left to right: beta sheet (blue), right alpha helix (red), left helix (green), unclassified (yellow). We use a different colorbar scale (normalized to the highest value) to visualize left α-helix and unclassified to make the patterns visible.

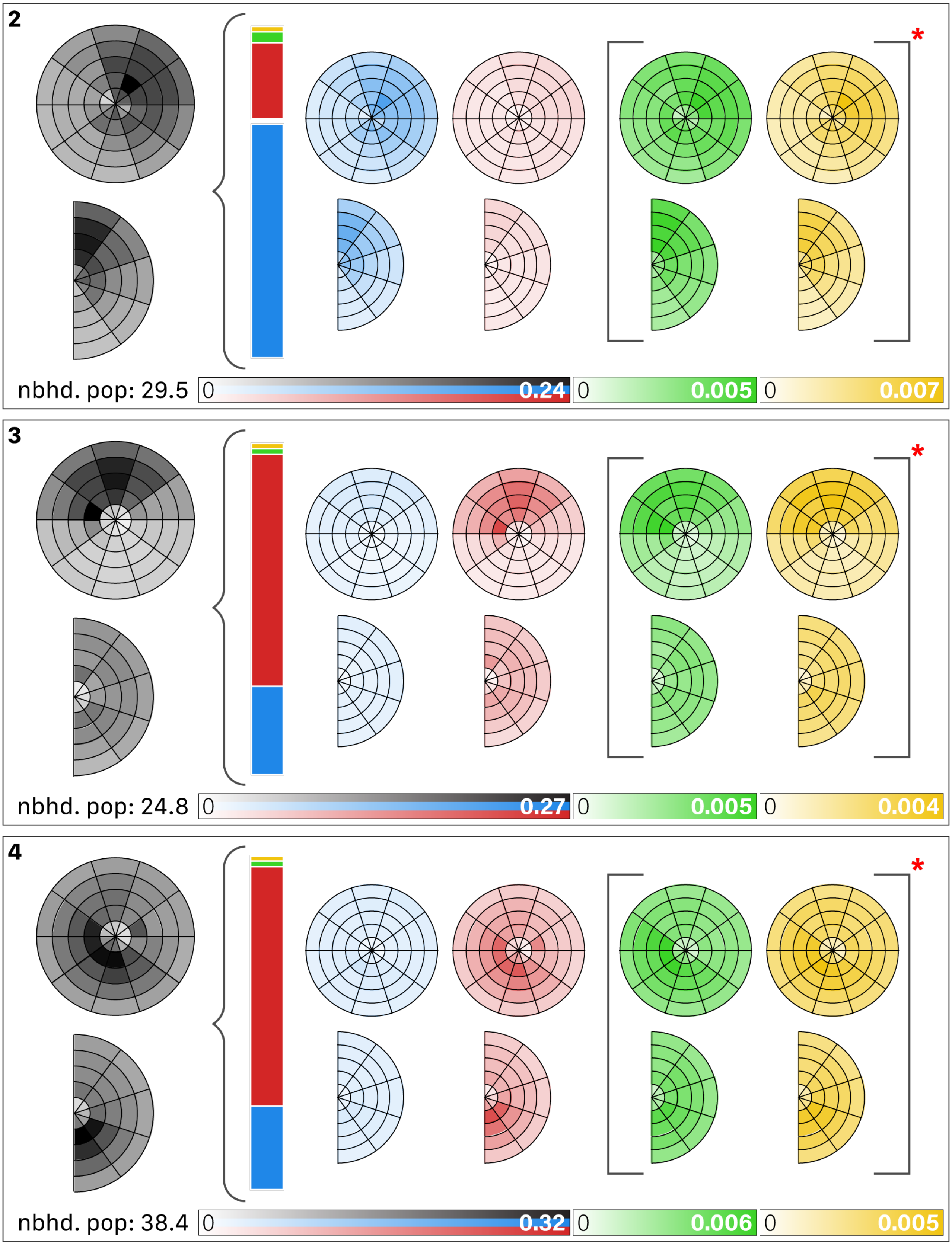
2
*
nbhd. pop: 29.5
0
0.24
0
0.005
0
0.007
3
*
nbhd. pop: 24.8
0
0.27
0
0.005
0
0.004
4
*
nbhd. pop: 38.4
0
0.32
0
0.006
0
0.005

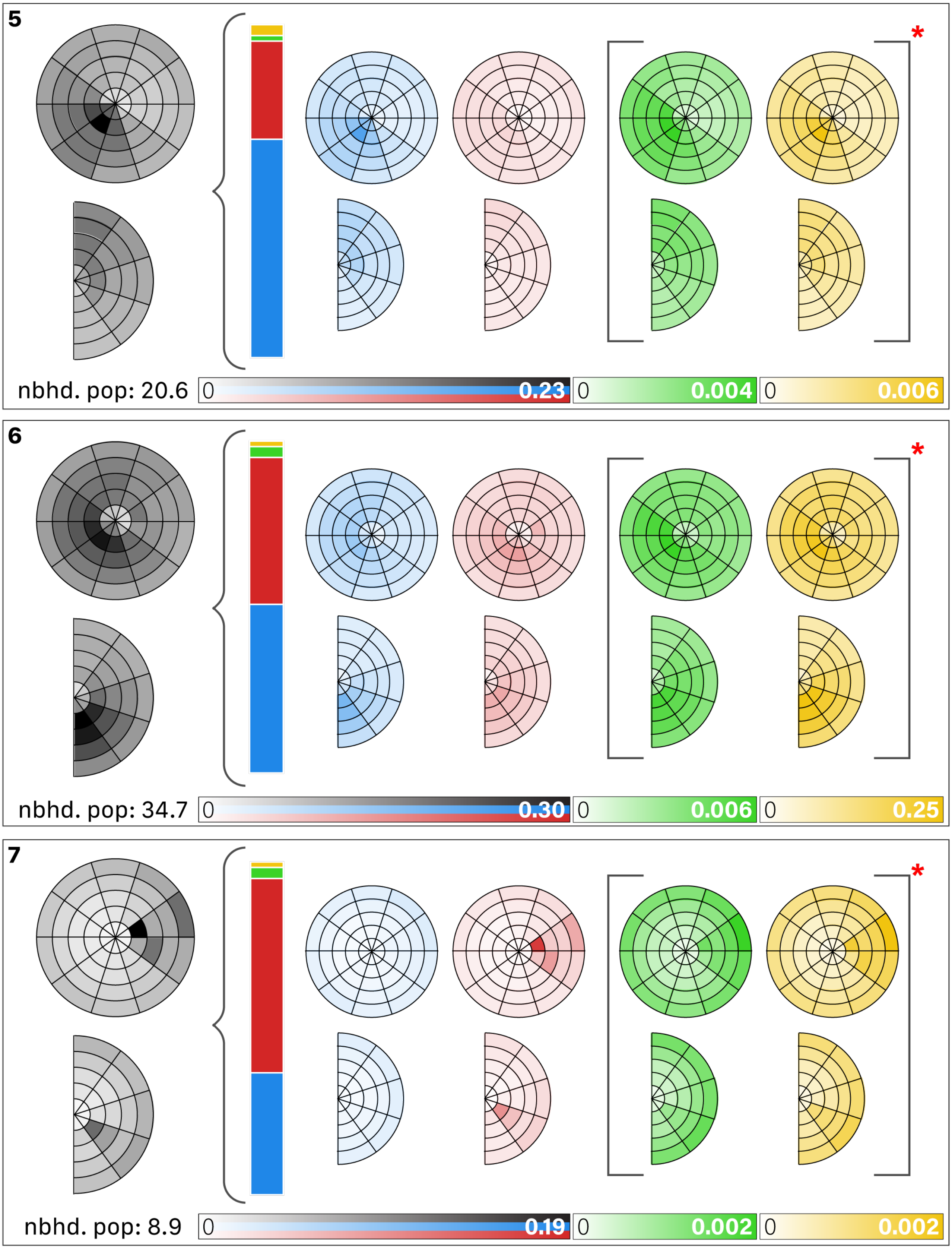
5
*
nbhd. pop: 20.6
0
0.23
0
0.004
0
0.006
6
*
nbhd. pop: 34.7
0
0.30
0
0.006
0
0.25
7
*
nbhd. pop: 8.9
0
0.19
0
0.002
0
0.002

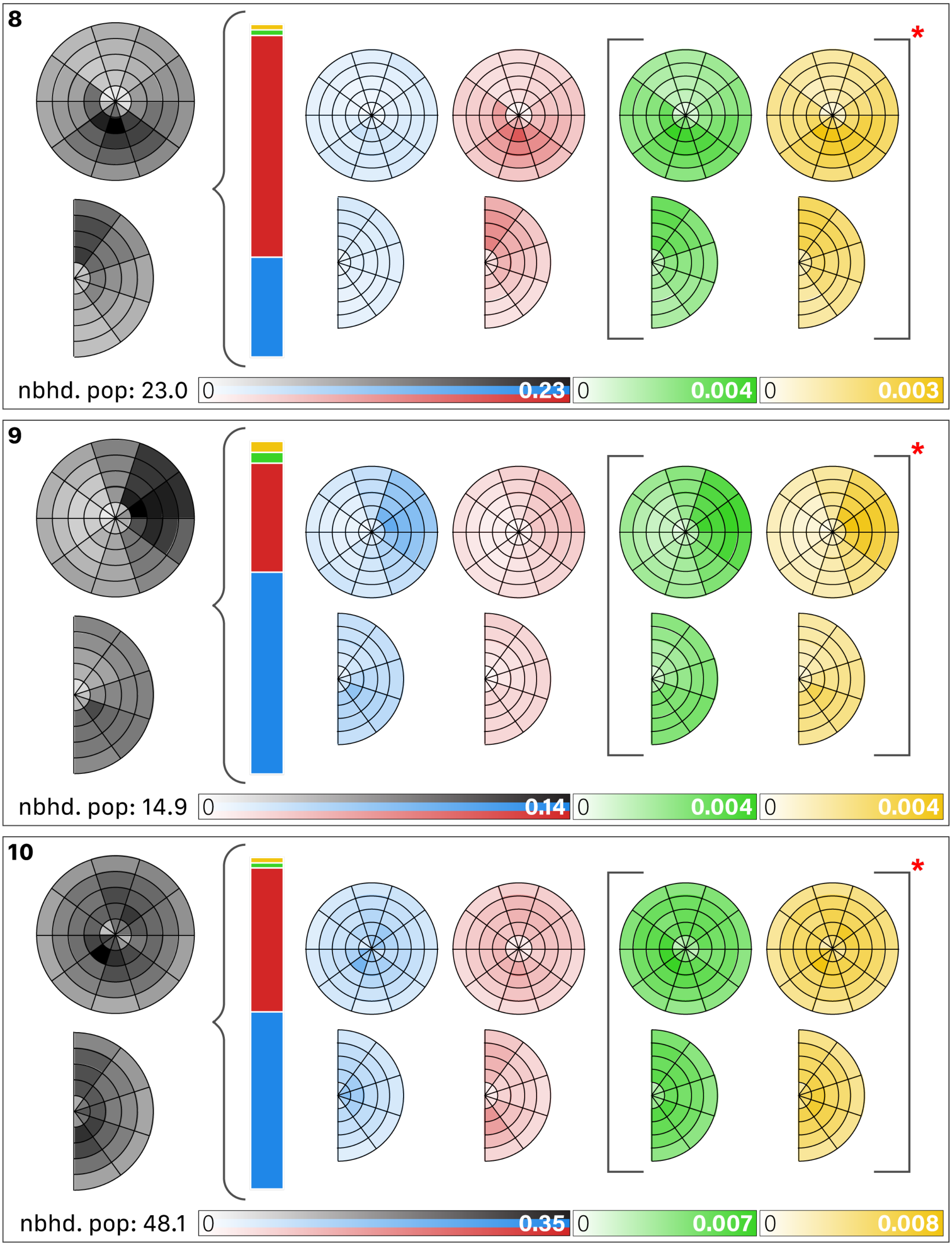

8
*
nbhd. pop: 23.0
0
0.23
0
0.004
0
0.003
9
*
nbhd. pop: 14.9
0
0.14
0
0.004
0
0.004
10
*
nbhd. pop: 48.1
0
0.35
0
0.007
0
0.008

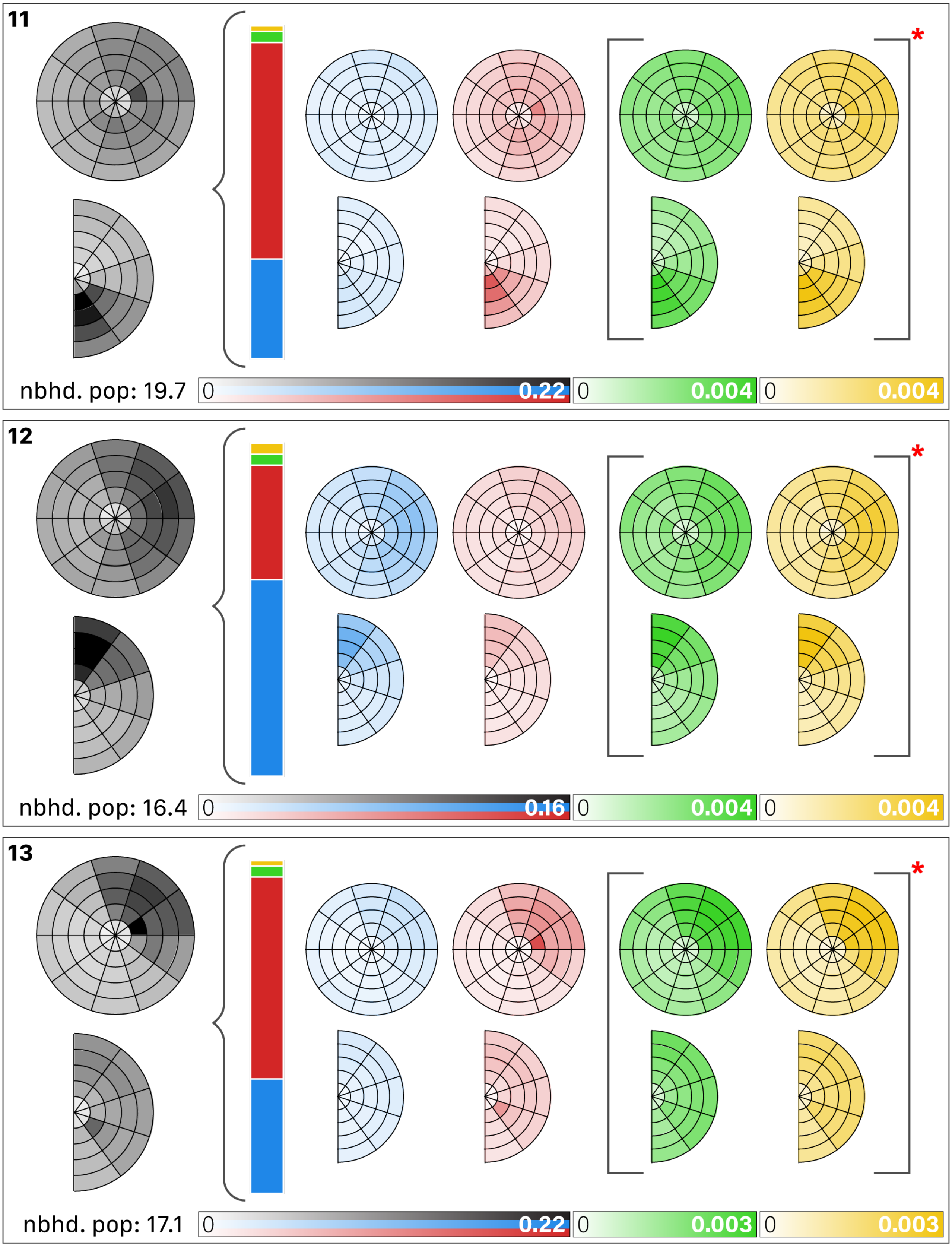
11
nbhd. pop: 19.7
0
0.22
0
0.004
0
0.004
12
nbhd. pop: 16.4
0
0.16
0
0.004
0
0.004
13
nbhd. pop: 17.1
0
0.22
0
0.003
0
0.003

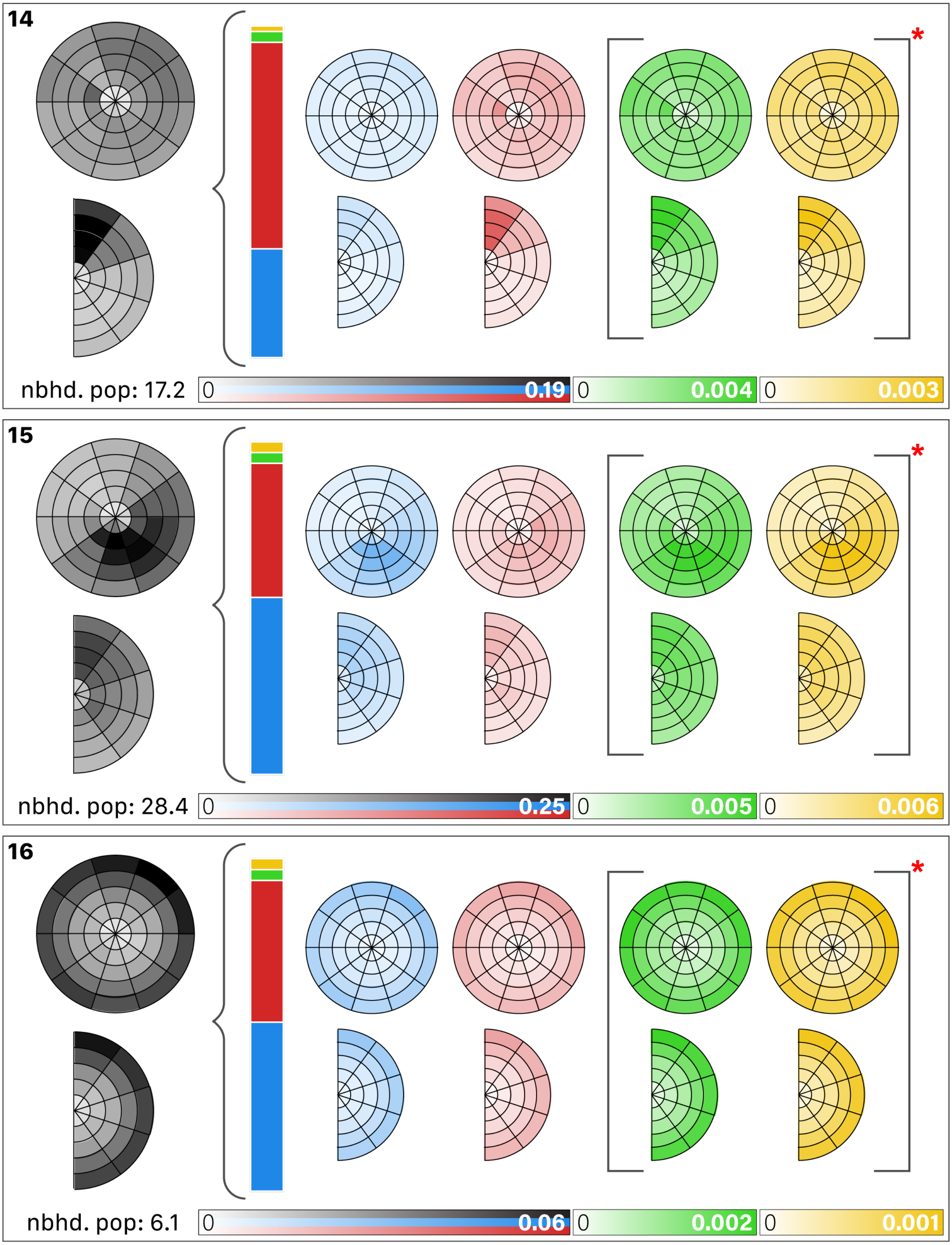
14
*
nbhd. pop: 17.2
0
0.19
0
0.004
0
0.003
15
*
nbhd. pop: 28.4
0
0.25
0
0.005
0
0.006
16
*
nbhd. pop: 6.1
0
0.06
0
0.002
0
0.001

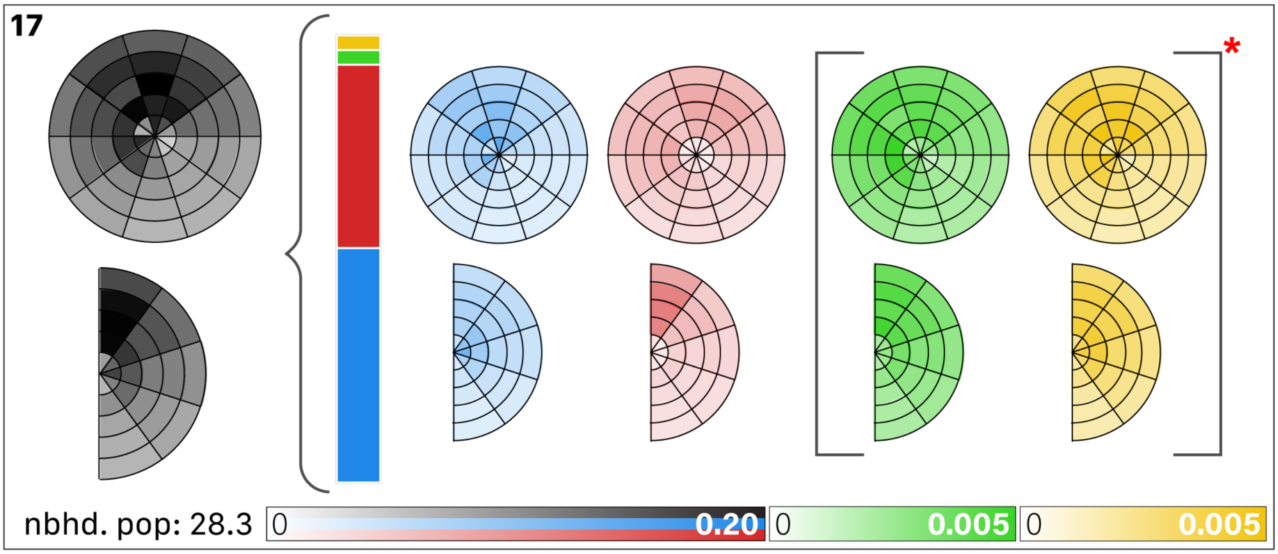
17
*
nbhd. pop: 28.3
0
0.20
0
0.005
0
0.005

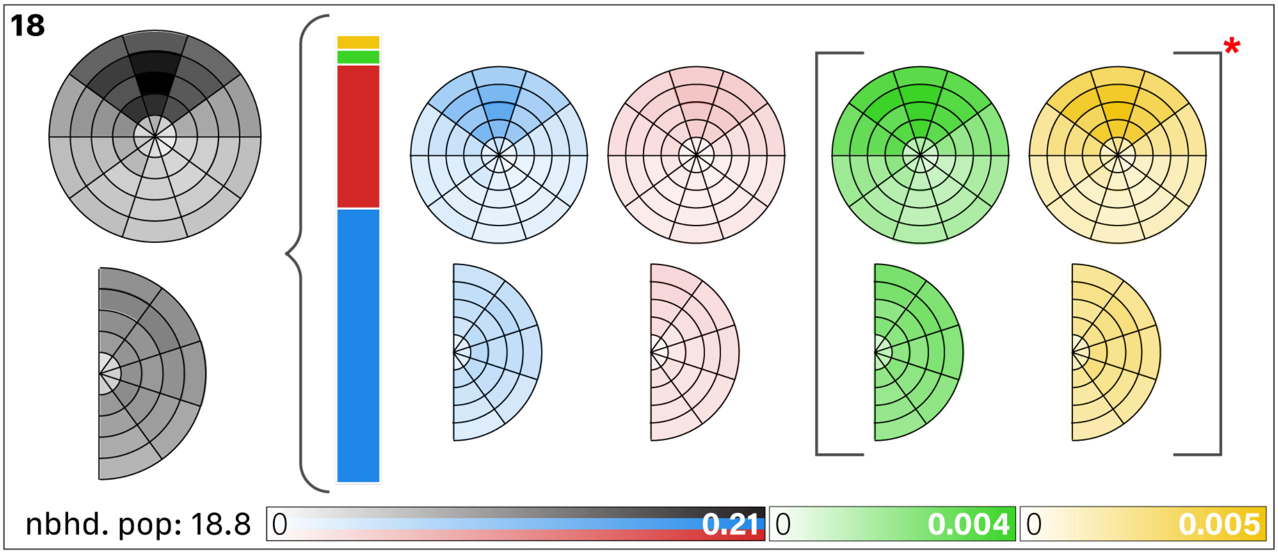
18
*
nbhd. pop: 18.8
0
0.21
0
0.004
0
0.005

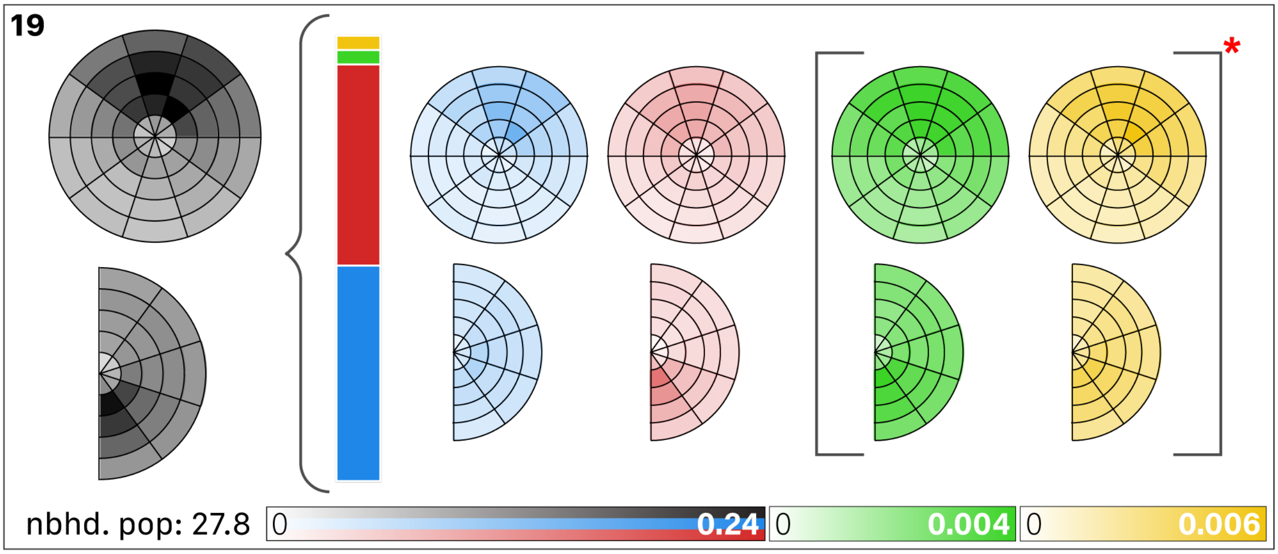
19
*
nbhd. pop: 27.8
0
0.24
0
0.004
0
0.006

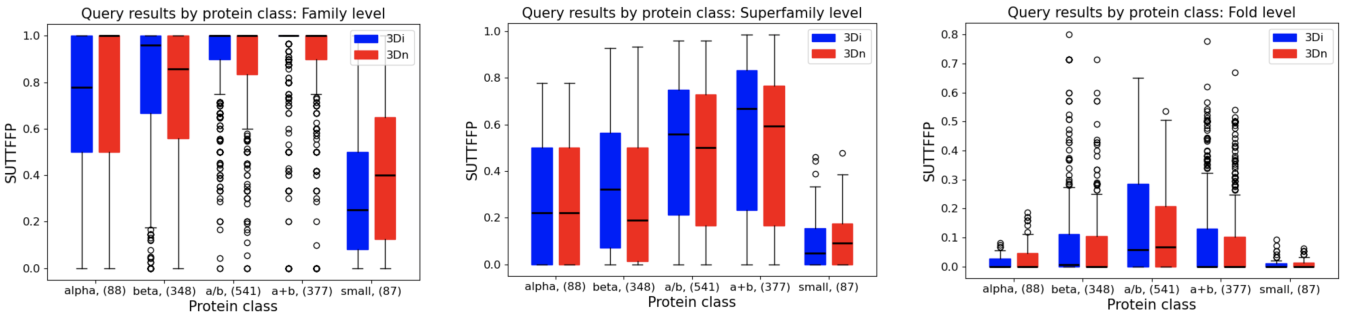

Fig. 13: **Query results by protein class at family, superfamily, and fold levels in the search benchmarking task.** Query results are quantified by sensitivity up to the first false positive (SUTTFFP). Protein class refers to the class of the SCOPe ID of the query protein: 'alpha' refers to proteins dominated by alpha helices, 'beta' refers to proteins dominated by beta sheets, 'a/b' refers to proteins that contain alternating alpha helix and beta sheet components within the protein, 'a+b' refers to proteins where alpha helix and beta sheet components exist in separate, continuous regions in the protein, and 'small' refers to small proteins. The number of queries in the class is noted in parentheses. In the box plot, the bottom edge of the box is at x_{25}, the upper edge is at x_{75}, the middle bar represents the median value x_{50}, the bottom whisker is at $\max(0, x_{25} - 1.5(x_{75} - x_{25}))$, and the top whisker is at $\min(1.0, x_{75} + 1.5(x_{75} - x_{25}))$, where x_p represents the value at percentile p.

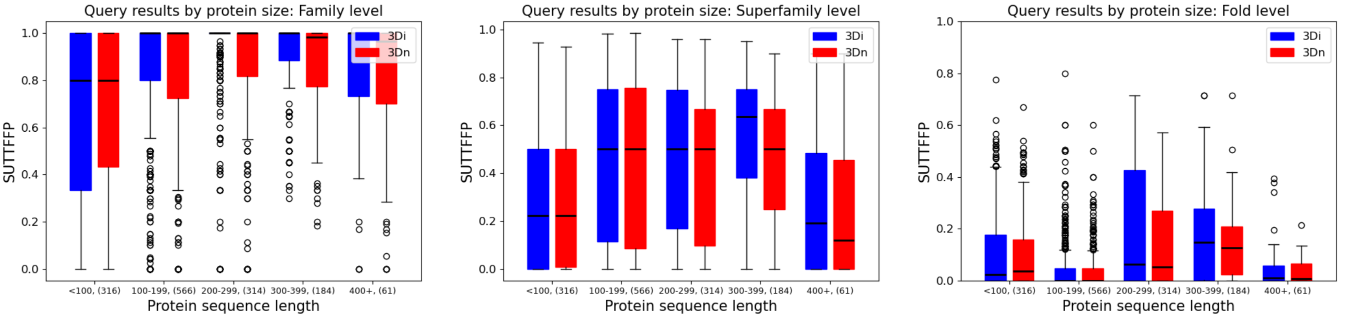

Fig. 14: **Query results by protein sequence length at family, superfamily, and fold levels in the search benchmarking task.** Query results are quantified by sensitivity up to the first false positive (SUTTFFP). Proteins are grouped into five categories based on sequence length. The number of queries in the protein length category is noted in parentheses. In the box plot, the bottom edge of the box is at x_{25}, the upper edge is at x_{75}, the middle bar represents the median value x_{50}, the bottom whisker is at $\max(0, x_{25} - 1.5(x_{75} - x_{25}))$, and the top whisker is at $\min(1.0, x_{75} + 1.5(x_{75} - x_{25}))$, where x_p represents the value at percentile p.

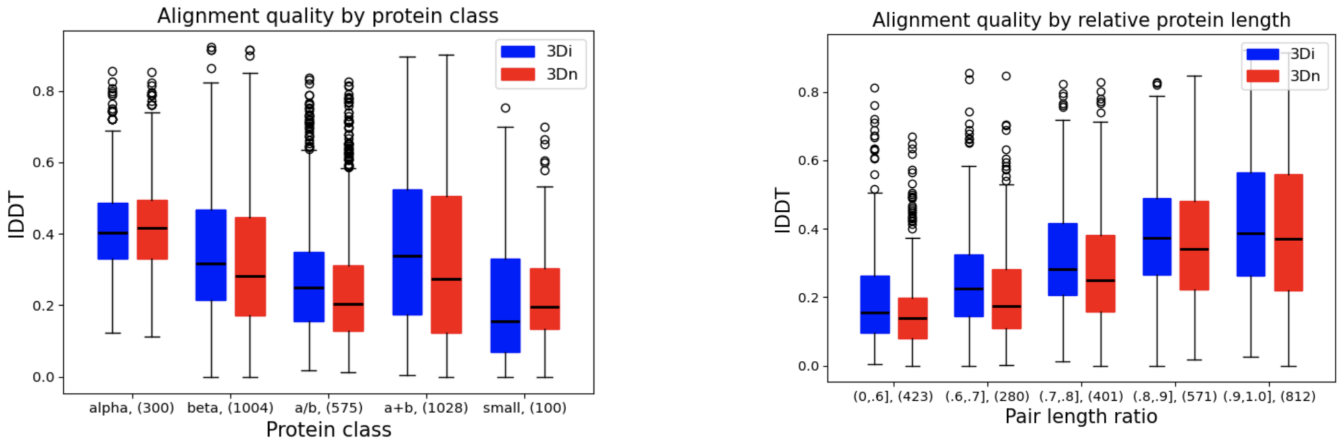

Fig. 15: **Alignment quality on the test set for 3Di vs. 3Dn with respect to various protein qualities.** The figure on the left depicts lDDT of protein pairs with respect to the class of the proteins within the pair, according to their SCOPe IDs:'alpha' refers to proteins dominated by alpha helices, 'beta' refers to proteins dominated by beta sheets, 'a/b' refers to proteins that contain alternating alpha helix and beta sheet components within the protein, 'a+b' refers to proteins where alpha helix and beta sheet components exist in separate, continuous regions in the protein, and 'small' refers to small proteins. The number of pairs in the class is noted in parentheses. The figure on the right depicts lDDT with respect to the ratio of the sequence length of the shorter protein in the pair to the sequence length of the longer protein in the pair. Each bar in the plot represents lDDT of pairs which length ratio lies in the corresponding interval indicated on the x-axis, and the number of pairs in the pair length classification is noted in parentheses. In the box plot, the bottom edge of the box is at x_{25}, the upper edge is at x_{75}, the middle bar represents the median value x_{50}, the bottom whisker is at $\max(0, x_{25} - 1.5(x_{75} - x_{25}))$, and the top whisker is at $\min(1.0, x_{75} + 1.5(x_{75} - x_{25}))$, where x_p represents the value at percentile p.
